# ZMYND8 protects breast cancer stem cells against oxidative stress and ferroptosis through activation of NRF2

**DOI:** 10.1172/JCI171166

**Published:** 2024-01-23

**Authors:** Maowu Luo, Lei Bao, Yuanyuan Xue, Ming Zhu, Ashwani Kumar, Chao Xing, Jennifer E. Wang, Yingfei Wang, Weibo Luo

**Affiliations:** 1 Department of Pathology,; 2 Children’s Medical Center Research Institute,; 3 Eugene McDermott Center for Human Growth and Development,; 4 Lyda Hill Department of Bioinformatics,; 5 Department of Neurology,; 6 Peter O’Donnell Jr. Brain Institute,; 7 Cecil H. and Ida Green Center for Reproductive Biology Sciences, and; 8 Department of Pharmacology, UT Southwestern Medical Center, Dallas, Texas, USA.

**Keywords:** Stem cells, Cell stress, Epigenetics, Transcription

## Abstract

Breast cancer stem cells (BCSCs) mitigate oxidative stress to maintain their viability and plasticity. However, the regulatory mechanism of oxidative stress in BCSCs remains unclear. We recently found that the histone reader ZMYND8 was upregulated in BCSCs. Here, we showed that ZMYND8 reduced ROS and iron to inhibit ferroptosis in aldehyde dehydrogenase–high (ALDH^hi^) BCSCs, leading to BCSC expansion and tumor initiation in mice. The underlying mechanism involved a two-fold posttranslational regulation of nuclear factor erythroid 2–related factor 2 (NRF2). ZMYND8 increased stability of NRF2 protein through KEAP1 silencing. On the other hand, ZMYND8 interacted with and recruited NRF2 to the promoters of antioxidant genes to enhance gene transcription in mammospheres. NRF2 phenocopied ZMYND8 to enhance BCSC stemness and tumor initiation by inhibiting ROS and ferroptosis. Loss of NRF2 counteracted ZMYND8’s effects on antioxidant genes and ROS in mammospheres. Interestingly, ZMYND8 expression was directly controlled by NRF2 in mammospheres. Collectively, these findings uncover a positive feedback loop that amplifies the antioxidant defense mechanism sustaining BCSC survival and stemness.

## Abstract 2

ZMYND8 negatively regulates ferroptosis in breast cancer stem cells.

## Graphical abstract

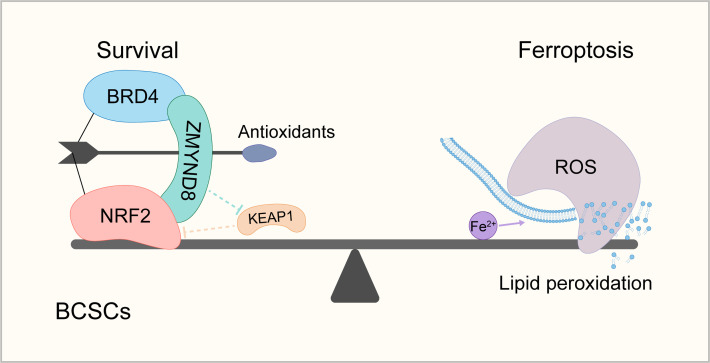

## Introduction

Breast cancer stem cells (BCSCs) are defined as a subpopulation of tumor cells with capability of self renewal and long-term maintenance of breast tumors and are responsible for breast tumor initiation, metastasis, and therapy resistance (1, 2). Thus, they are considered as the root of the malignant disease. Understanding how BCSC fate is regulated is not only important for gaining mechanistic insights into the maintenance of BCSCs but also for the development of effective therapeutic approaches to treat human breast cancer.

Ferroptosis is a nonapoptotic form of cell death driven by iron-dependent lipid peroxidation and associated with the perturbation of the redox homeostasis (3, 4). Emerging evidence has shown that ferroptosis plays a critical role in BCSC fate. The concentration of iron is higher in cancer stem cells (CSCs) than in non-CSCs (5), suggesting that CSCs are highly susceptible to ferroptosis. A recent study reported that the intrinsic plastic phenotype protects BCSCs against ferroptosis (6). However, the signaling regulatory mechanism of ferroptosis in BCSCs remains poorly defined.

Redox homeostasis is critical for ferroptosis and CSC fate (7). CSCs express the high levels of antioxidants to maintain the low levels of ROS (8). Nuclear factor erythroid 2–related factor 2 (NRF2) is a master transcriptional regulator of redox homeostasis and its hyperactivation promotes tumor progression, metastasis, and resistance to therapies (8–10). NRF2 heterodimerizes with small musculoaponeurotic fibrosarcoma proteins and binds to the antioxidant response element (ARE) at the promoter of its target genes leading to the transcription of antioxidant genes (8, 10, 11). The transcriptional activity of NRF2 is primarily regulated at the protein level through a proteasomal degradation mechanism (11). Multiple ubiquitin E3 ligases, including the canonical Kelch-like ECH-associated protein 1–Cullin3 (KEAP1–CUL3) E3 ligase complex that binds the DLG and ETGE motifs of NRF2, the β-transducin repeat-containing protein Cullin1–Rbx1 E3 ligase complex, the WD repeat-containing protein 23-DDB1–Cullin4 E3 ligase complex, and the E3 ubiquitin ligase HMG–CoA reductase degradation 1 homolog, have been shown to control NRF2 protein stability (10, 11). Hyperactivation of NRF2 can occur in cancer cells through diverse mechanisms, including somatic mutations of *NFE2L2* (mainly within the DLG and ETGE motifs), *KEAP1*, or *CUL3* genes; transcriptional upregulation of *NFE2L2* by oncogenes (e.g., *MYC*, *KRAS*^G12D^, and *BRAF*^V619E^); accumulation of KEAP1-associated proteins (e.g., p62); and epigenetic alterations of the *KEAP1* and *NFE2L2* promoters (10, 11). However, epigenetic regulation of NRF2 transcriptional activity has not been well studied.

Zinc finger MYND-type containing 8 (ZMYND8) is a histone reader protein recognizing acetyl-lysine 16 of histone H4 (H4K16ac), acetyl-lysine 14 of histone H3, monomethyl lysine 4 of histone H3, di- or trimethyl lysine 36 of histone H3, and histone H3.3G34R via a reader cassette containing plant homeodomain, bromodomain, and Pro-Trp-Trp-Pro domain at its N-terminus (12–14). ZMYND8 coactivates genes by cooperating with hypoxia-inducible factor–1/2α, BRD4, and P-TEFb complex (15, 16) and also interacts with KDM5C, NuRD complex, and EZH2 for gene silencing (14, 17, 18). ZMYND8 is also involved in genome stability to block the cyclic GMP–AMP synthase-stimulator of interferon genes–NF-κB signaling and the subsequent interferon-β production in breast cancer cells, leading to cancer cell evasion from cytotoxic T-lymphocyte surveillance (19). Our recent study has identified a role of ZMYND8 in maintenance of BCSCs and breast tumor initiation (12). In addition to its role in breast cancer, ZMYND8 is upregulated and promotes tumor growth in multiple cancer models, including acute myeloid leukemia, bladder cancer, clear cell renal cell carcinoma, colorectal cancer, glioblastoma, and liver cancer (13, 15–17, 19–23).

In this study, we showed that ZMYND8 attenuated oxidative stress and ferroptosis in aldehyde dehydrogenase–high (ALDH^hi^) BCSCs to promote tumor initiation in vitro and in mice. ZMYND8 increased NRF2 protein stability and physically bound NRF2 to enhance the expression of NRF2-dependent antioxidant genes in BCSCs. We also found that ZMYND8 expression was directly controlled by NRF2 in BCSCs. Collectively, we believe that our findings uncover a mutual regulatory mechanism that protects BCSCs against oxidative stress and ferroptosis.

## Results

### ZMYND8 reduces oxidative stress in ALDH^hi^ BCSCs.

**Figure 1:**
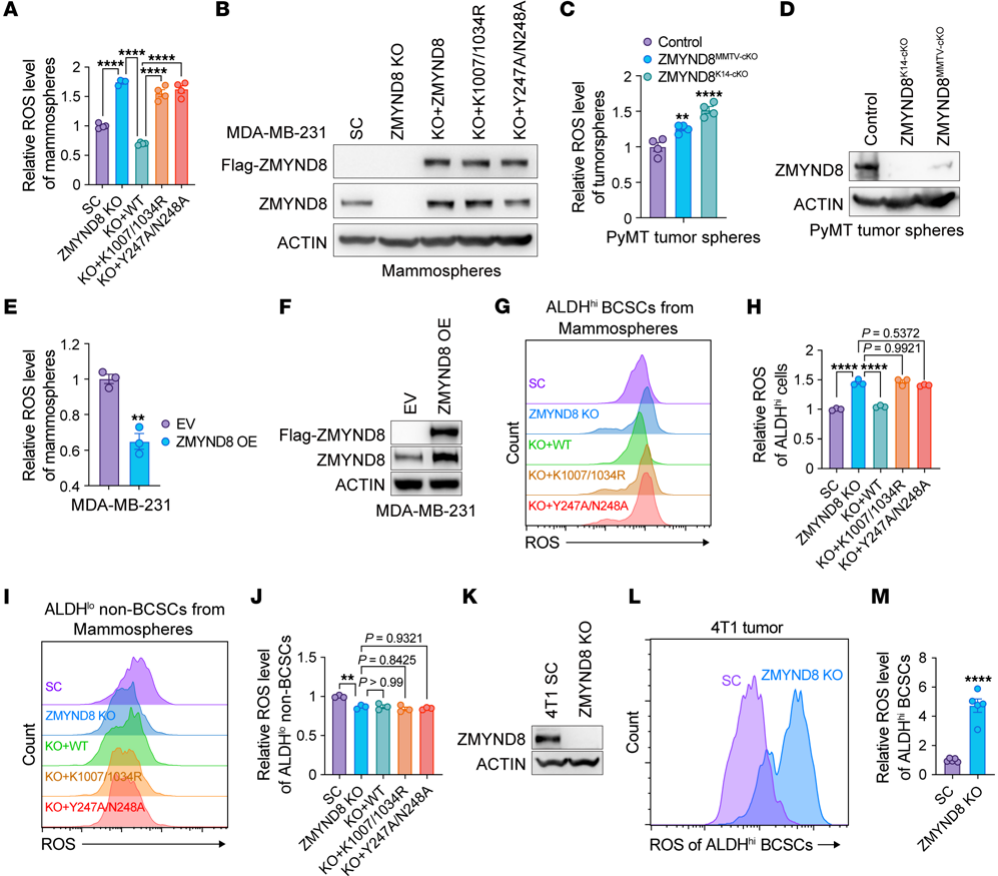
ZMYND8 reduces oxidative stress in ALDH^hi^ BCSCs. (**A**) Relative ROS levels in MDA-MB-231-SC, ZMYND8 KO11, and ZMYND8 KO rescued with WT ZMYND8, K1007/1034R, or Y247A/N248A mammospheres (*n* = 4). (**B**) Analysis of ZMYND8 and Flag-ZMYND8 protein levels in MDA-MB-231-SC, ZMYND8 KO, and ZMYND8 KO rescued with WT ZMYND8, K1007/1034R, or Y247A/N248A mammospheres (*n* = 3). (**C**) Relative ROS levels in tumorspheres from control (*MMTV-PyMT*^–/+^ and *Zmynd8*^+/+^), ZMYND8^MMTV–cKO^ (*MMTV-PyMT*^–/+^, *MMTV-Cre*^–/+^, and *Zmynd8^fl/fl^*), and ZMYND8^K14–cKO^ (*MMTV-PyMT*^–/+^, *K14-Cre*^–/+^, and *Zmynd8^fl/fl^*) mammary tumors (*n* = 4). (**D**) Analysis of ZMYND8 protein levels in tumorspheres from control (*MMTV-PyMT*^–/+^ and *Zmynd8*^+/+^), ZMYND8^MMTV–cKO^ (*MMTV-PyMT*^–/+^, *MMTV-Cre*^–/+^, and *Zmynd8^fl/fl^*), and ZMYND8^K14–cKO^ (*MMTV-PyMT*^–/+^, *K14-Cre*^–/+^, and *Zmynd8^fl/fl^*) mammary tumors (*n* = 4). (**E**) Relative ROS levels in MDA-MB-231 mammospheres expressing EV or WT ZMYND8 (*n* = 3). (**F**) Analysis of ZMYND8 protein levels in MDA-MB-231 mammospheres expressing EV or WT ZMYND8 (*n* = 3). (**G**–**J**) Relative ROS levels in ALDH^hi^ BCSCs (**G** and **H**) and ALDH^lo^ non-BCSCs (**I** and **J**) cells from MDA-MB-231-SC, ZMYND8 KO, and ZMYND8 KO rescued with WT ZMYND8, K1007/1034R, or Y247A/N248A mammospheres (*n* = 3). (**K**) Analysis of ZMYND8 protein levels in SC and ZMYND8 KO 4T1 cells (*n* = 3). (**L** and **M**) Relative ROS levels in ALDH^hi^ BCSCs from SC and ZMYND8 KO 4T1 allograft tumors (*n* = 5). Data represent mean ± SEM. *P* values determined by using 1-way ANOVA corrected with Tukey’s test (**A**, **H**, and **J**), Dunnett’s test (**C**), or 2-tailed Student’s *t* test (**E** and **M**). **P < 0.01; ****P < 0.0001.

We recently found that ZMYND8 was upregulated to induce genes involved in ROS and xenobiotic metabolism in BCSCs (12). To assess whether ZMYND8 controls ROS homeostasis in BCSCs, we quantified cellular ROS with a cell-permeable ROS probe, 2′,7′-dichlorofluorescin diacetate, and found that ROS was significantly increased in ZMYND8-KO MDA-MB-231 mammospheres, which was abolished by reexpression of WT ZMYND8 but not BRD4 binding site mutant K1007/1034R or H4K16ac binding site mutant Y247A/N248A (Figure 1, A and B). Similar results were observed in MCF-7 mammospheres following ZMYND8 KO (Supplemental Figure 1, A and B; supplemental material available online with this article; https://doi.org/10.1172/JCI171166DS1). We further showed that deletion of ZMYND8 with MMTV-Cre or K14-Cre significantly increased ROS in tumorspheres prepared from MMTV-PyMT mammary tumors ex vivo (Figure 1, C and D). In contrast, over expression (OE) of ZMYND8 significantly decreased the cellular ROS levels in both MDA-MB-231 and MCF-7 mammospheres (Figure 1, E and F and Supplemental Figure 1, C and D). To assess ROS regulation by ZMYND8 in a specific BCSC population, we measured cellular ROS levels with flow cytometry in ALDH^hi^ BCSCs and ALDH^lo^ non-BCSCs from cultured MDA-MB-231 mammospheres or monolayer cells. ZMYND8 reduced ROS through its epigenetic activities in ALDH^hi^ BCSCs isolated from both mammospheres and monolayers (Figure 1, G and H and Supplemental Figure 1, E and F). In line with K1007/1034R mutant, treatment of the BET inhibitor JQ1 (0.5 μM) induced ROS in ALDH^hi^ BCSCs (Supplemental Figure 1G). In contrast, ZMYND8 did not affect ROS levels in ALDH^lo^ non-BCSCs isolated from MDA-MB-231 mammospheres and monolayers (Figure 1, I and J and Supplemental Figure 1, H and I). We also detected increased ROS in ALDH^hi^ BCSCs derived from ZMYND8 KO1 or KO2 MCF-7 monolayers (Supplemental Figure 1, J and K). Lastly, we confirmed increased ROS in ALDH^hi^ BCSCs isolated from ZMYND8 KO 4T1 allograft tumors (Figure 1, K–M). Together, these results indicate that ZMYND8 is a negative regulator of ROS in ALDH^hi^ BCSCs.

### ZMYND8 increases maintenance of ALDH^hi^ BCSCs by inhibiting ROS.

**Figure 2:**
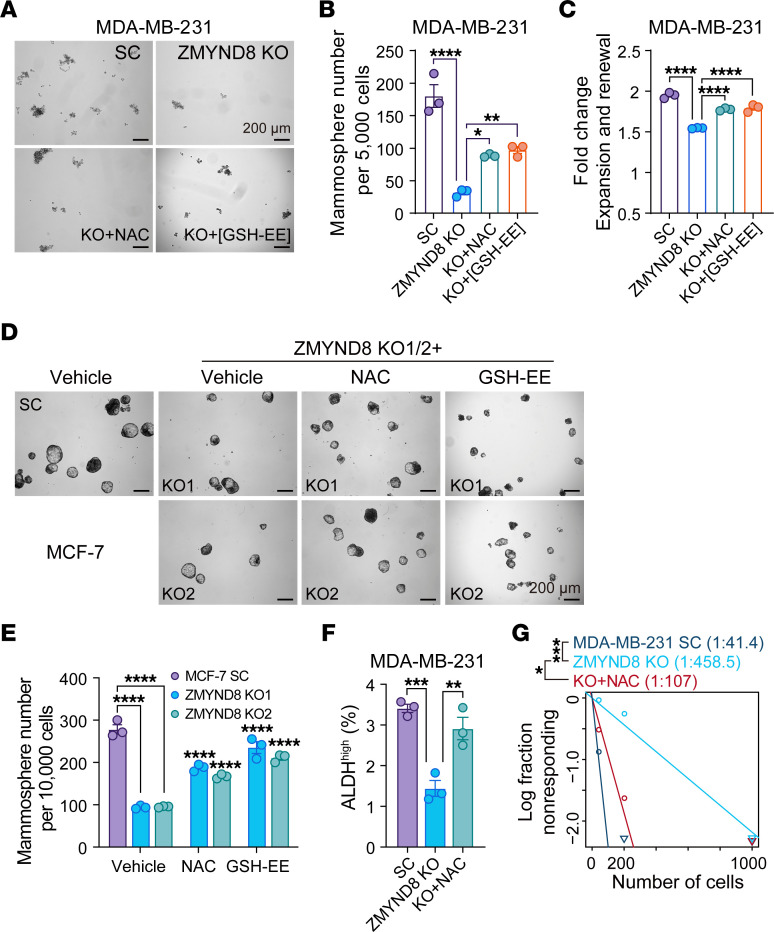
ZMYND8 promotes maintenance of ALDH^hi^ BCSCs through reducing ROS. (**A**–**C**) Formation of MDA-MB-231-SC, ZMYND8 KO, and ZMYND8 KO mammospheres treated with vehicle, NAC, or GSH-EE. Representative mammosphere images (**A**). Quantification of primary mammosphere numbers (**B**) and mammosphere expansion and self-renewal (**C**, *n* = 3). (**D** and **E**) Formation of MCF-7-SC, ZMYND8 KO1, and ZMYND8 KO2 mammospheres treated with DMSO, NAC, or GSH-EE. Representative mammosphere images (**D**). Quantification of primary mammosphere numbers (**E**, *n* = 3). (**F**) Quantification of ALDH^hi^ BCSCs in MDA-MB-231-SC and ZMYND8 KO cells treated with or without NAC (*n* = 3). (**G**) Limiting dilution assay of MDA-MB-231-SC and ZMYND8 KO cells in NSG mice (*n* = 5). Mice implanted with ZMYND8 KO cells were fed with regular or NAC water. Data represent mean ± SEM. *P* values were determined by using 1-way ANOVA corrected with Tukey’s test (**B**, **C**, and **F**) or 2-way ANOVA corrected with Tukey’s test (**E**), and κ^2^ test (**G**). *P < 0.05; **P < 0.01; ***P < 0.001; ****P < 0.0001. Scale bars: 200 μm.

To determine a role of ROS in ZMYND8-induced BCSC stemness, we treated ZMYND8 KO MDA-MB-231 mammospheres with an antioxidant *N*-acetyl-*L*-cysteine (NAC) or glutathione ethyl ester (GSH-EE). As expected, ZMYND8 KO inhibited MDA-MB-231 mammosphere formation and self renewal (Figure 2, A–C). Clearance of ROS with NAC or GSH-EE partially prevented the loss of BCSC stemness conferred by ZMYND8 KO (Figure 2, A–C). Similar results were observed in MCF-7 mammospheres (Figure 2, D and E). We further showed that ZMYND8 KO decreased the number of ALDH^hi^ BCSCs, which was blocked by NAC treatment (Figure 2F). In contrast, ZMYND8 OE significantly increased the formation of MDA-MB-231 and MCF-7 mammospheres (Supplemental Figure 2, A–D). Treatment with JQ1 (0.5 μM) counteracted ZMYND8 OE to inhibit mammosphere formation (Supplemental Figure 2, A–D), consistent with a role of BRD4 in ZMYND8-suppressed ROS (Figure 1A and Supplemental Figure 1G). Next, we investigated whether ZMYND8 reduced ROS to increase mammary tumor initiation in vivo by limiting dilution assay. Scrambled control (SC) and ZMYND8-KO MDA-MB-231 cells with 3 serial dilutions were orthotopically implanted into the mammary fat pad of female NOD-scid IL2R-γ^null^ (NSG) mice, respectively, and mice were fed with regular water or NAC-containing water. NAC water feeding significantly restored the ZMYND8-KO tumor incidence in mice (Figure 2G). Collectively, these results indicate that ZMYND8 maintains ALDH^hi^ BCSC stemness in vitro and in vivo by reducing ROS.

Our previous studies showed that ZMYND8 blocks T-lymphocyte surveillance to promote breast tumor growth (19). To exclude a role of ZMYND8–mediated immune response in tumor initiation, we compared the initiation frequency of SC and ZMYND8-KO 4T1 tumors in both immunocompromised NSG mice and immunocompetent BALB/c mice using limiting dilution assay. ZMYND8 KO equally reduced the frequency of 4T1 tumor initiation in NSG and BALB/c mice (Supplemental Figure 3A). However, 4T1 tumor regression by ZMYND8 KO was much greater in BALB/c mice than NSG mice (Supplemental Figure 3, B and C). These results indicate that ZMYND8-mediated immune evasion contributes to tumor progression but not tumor initiation in mice.

### ZMYND8 protects ALDH^hi^ BCSCs from oxidative stress–induced ferroptosis.

**Figure 3:**
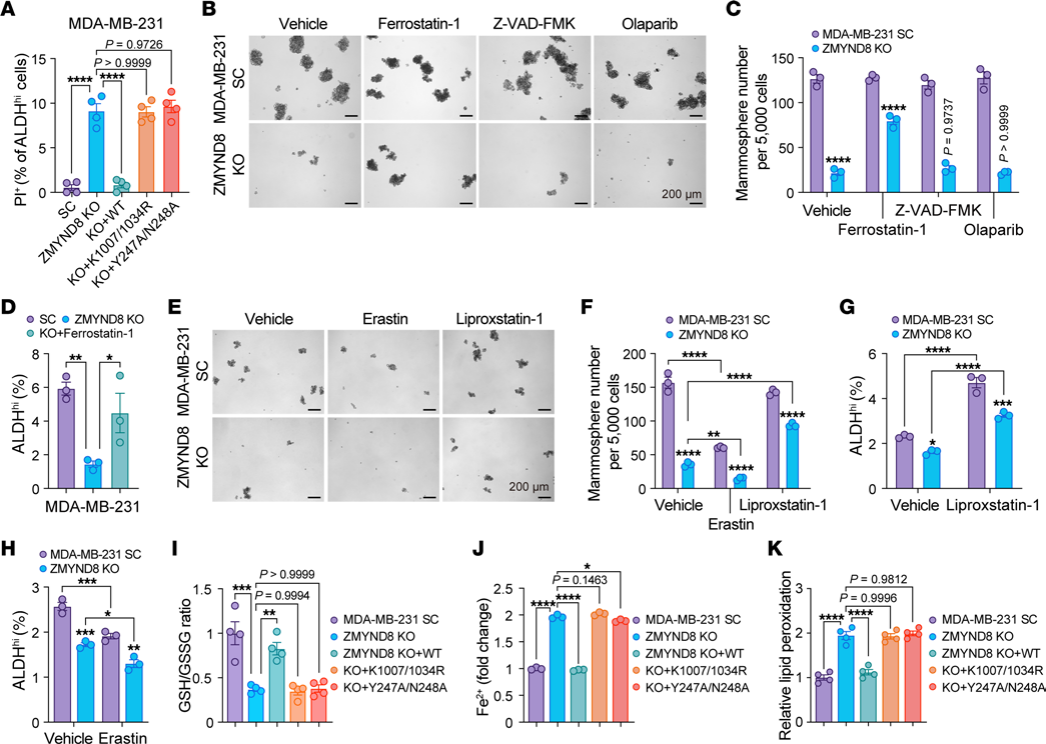
ZMYND8 inhibits ferroptosis in ALDH^hi^ BCSCs. (**A**) Death of ALDH^hi^ BCSCs in MDA-MB-231-SC, ZMYND8 KO, and ZMYND8 KO rescued with WT ZMYND8, K1007/1034R, or Y247A/N248A mammospheres (*n* = 4). (**B** and **C**) Formation of MDA-MB-231-SC, ZMYND8 KO, and ZMYND8 KO mammospheres treated with vehicle, Ferrostatin-1, Z-VAD-FMK, or Olaparib. Representative mammosphere images (**B**). Quantification of mammosphere numbers (**C**, *n* = 3). (**D**) Quantification of ALDH^hi^ BCSCs in MDA-MB-231-SC and ZMYND8 KO cells treated with or without Ferrostatin-1 (*n* = 3). (**E** and **F**) Formation of MDA-MB-231-SC, ZMYND8 KO, and ZMYND8 KO mammospheres treated with vehicle, Erastin, or Liproxstatin-1. Representative mammosphere images (**E**). Quantification of mammosphere numbers (**F**, *n* = 3). (**G** and **H**) Quantification of ALDH^hi^ BCSCs in MDA-MB-231-SC and ZMYND8 KO cells treated with or without Liproxstatin-1 (**G**) or Erastin (**H**, *n* = 3). (**I**–**K**) Quantification of GSH/GSSG (**I**), Fe^2+^ (**J**), and lipid peroxidation (**K**) in MDA-MB-231-SC, ZMYND8 KO, ZMYND8 KO rescued with WT ZMYND8, K1007/1034R, or Y247A/N248A mammospheres (*n* = 3-4). Data represent mean ± SEM. *P* value was determined by using 1-way ANOVA corrected with Tukey’s test (**A**, **D**, **I**–**K**) or 2-way ANOVA corrected with Tukey’s test (**C**, **F**, **G**, and **H**). *P < 0.05; **P < 0.01; ***P < 0.001; ****P < 0.0001. Scale bars: 200 μm.

Excessive ROS production causes oxidative damage to cell components including membrane lipids, proteins, and DNA leading to cell death through ferroptosis, apoptosis, and PARthanatos (8). We found by propidium iodide (PI) staining that ZMYND8 KO significantly increased death of ALDH^hi^ BCSCs isolated from MDA-MB-231 mammospheres, which was reversed by reexpression of WT ZMYND8 but not K1007/1034R or Y247A/N248A mutants (Figure 3A). We next assessed which type of cell death was regulated by ZMYND8 in ALDH^hi^ BCSCs and whether ZMYND8 inhibits cell death to increase ALDH^hi^ BCSC stemness. SC and ZMYND8-KO MDA-MB-231 mammospheres were treated with vehicle, ferroptosis inhibitor (Ferrostatin-1, 2.5 μM), apoptosis inhibitor (Z-VAD-FMK, 20 μM), or PARthanatos inhibitor (Olaparib, 10 μM). Ferrostatin-1 treatment partially prevented the loss of MDA-MB-231 mammospheres and ALDH^hi^ BCSCs conferred by ZMYND8 KO (Figure 3, B-D). A similar rescue effect was observed in ZMYND8 KO1 or KO2 MCF-7-derived mammospheres and ALDH^hi^ BCSCs after treatment with NAC or Ferrostatin-1 (Supplemental Figure 4, A–C). Another ferroptosis inhibitor Liproxstatin-1 had a rescue effect similar to Ferrostatin-1 (Figure 3, E–G and Supplemental Figure 4, D–F), whereas an activator of ferroptosis, Erastin, synergized with ZMYND8 KO to reduce mammosphere formation and ALDH^hi^ BCSC populations (Figure 3, E, F, and H and Supplemental Figure 4, D-F). Z-VAD-FMK and Olaparib failed to rescue the formation of ZMYND8-KO MDA-MB-231 mammospheres (Figure 3, B and C), which excludes a role of ZMYND8 in apoptosis and PARthanatos. To confirm that ZMYND8 regulated ferroptosis in BCSCs, we quantified cellular glutathione levels by fluorometric assay, cellular ferrous iron (Fe^2+^) by colorimetric assay, and lipid peroxidation by malondialdehyde assay. ZMYND8 KO significantly decreased the ratio of reduced and oxidized glutathione (GSH/GSSG), but increased Fe^2+^ levels in MDA-MB-231 mammospheres (Figure 3, I and J). In both MDA-MB-231 and MCF-7 mammospheres, lipid peroxidation was induced following ZMYND8 KO (Figure 3K and Supplemental Figure 4G). Reexpression of WT ZMYND8, but not K1007/1034R or Y247A/N248A mutant, increased GSH/GSSG, but reduced Fe^2+^ and lipid peroxidation levels in ZMYND8-KO MDA-MB-231 mammospheres (Figure 3, I-K). Together, these results indicate that ZMYND8 inhibits ferroptosis to maintain ALDH^hi^ BCSC stemness.

### ZMYND8 increases ROS clearance through activation of NRF2 in BCSCs.

**Figure 4:**
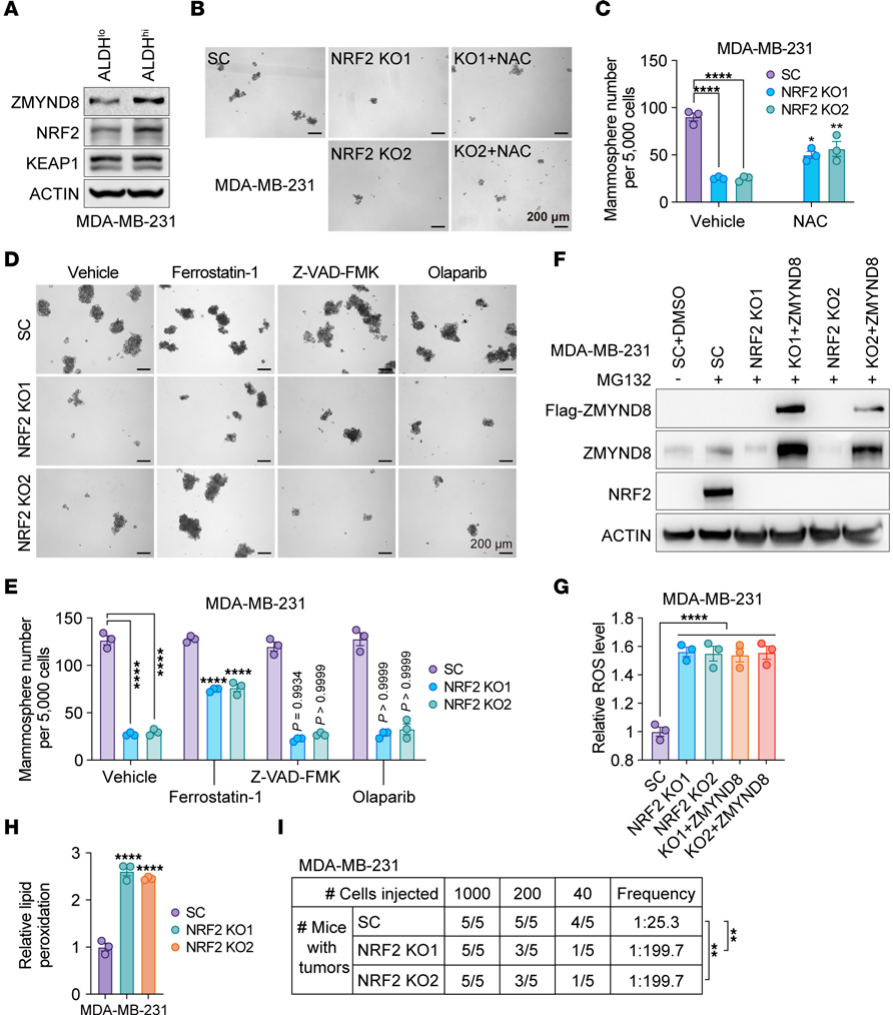
ZMYND8 inhibits ferroptosis in an NRF2-dependent manner. (**A**) Analysis of ZMYND8, NRF2, and KEAP1 protein levels in ALDH^hi^ BCSCs and ALDH^lo^ non-BCSCs from MDA-MB-231 cells (*n* = 3). (**B** and **C**) Formation of MDA-MB-231-SC, NRF2 KO1, and NRF2 KO2 mammospheres treated with or without NAC. Representative mammosphere images (**B**). Quantification of mammosphere numbers (**C**, *n* = 3). (**D** and **E**) Formation of MDA-MB-231-SC and NRF2 KO1/2 mammospheres treated with vehicle, Ferrostatin-1, Z-VAD-FMK, or Olaparib. Representative mammosphere images (**D**). Quantification of mammosphere numbers (**E**, *n* = 3). The experiments in Figures 3B and 4D were carried out concomitantly with the same MDA-MB-231-SC controls. (**F**) Analysis of ZMYND8 and NRF2 protein levels in MDA-MB-231-SC, NRF2 KO1/2, and NRF2 KO1/2 plus WT ZMYND8 cells treated with DMSO (–) or MG132 (+). *n* = 3. (**G**) Relative ROS levels in MDA-MB-231-SC, NRF2 KO1/2, and NRF2 KO1/2 plus WT ZMYND8 mammospheres (*n* = 3). (**H**) Relative lipid peroxidation levels in MDA-MB-231-SC and NRF2 KO1/2 mammospheres (*n* = 3). (**I**) Limiting dilution assay of MDA-MB-231-SC or NRF2 KO1/2 cells in NSG mice (*n* = 5). Data represent mean ± SEM. *P* value was determined by using 2-way ANOVA corrected with Tukey’s test (**C** and **E**), 1-way ANOVA corrected with Tukey’s test (**G**) or Dunnett’s test (**H**), and κ^2^ test (**I**). **P < 0.01; ****P < 0.0001. Scale bars: 200 μm.

The transcription factor NRF2 plays a central role against xenobiotics and ROS (8, 10, 11). Similar to ZMYND8, NRF2 protein was upregulated in MDA-MB-231 and MCF-7 mammospheres compared with their respective monolayers (Supplemental Figure 5, A and B). Upregulation of NRF2 and ZMYND8 proteins was also found in ALDH^hi^ BCSCs compared with ALDH^lo^ non-BCSCs, whereas KEAP1 protein levels were not changed (Figure 4A and Supplemental Figure 5C). NRF2 KO significantly decreased the formation of MDA-MB-231 and MCF-7 mammospheres, which was partially prevented by 1 mM NAC treatment (Figure 4, B and C and Supplemental Figure 5, D and E). Likewise, treatment of Ferrostatin-1 (2.5 μM), but not Z-VAD-FMK and Olaparib, partially rescued NRF2 KO mammospheres (Figure 4, D and E and Supplemental Figure 5, F and G). ROS and lipid peroxidation were significantly increased by NRF2 KO1 or KO2 in MDA-MB-231 and MCF-7 mammospheres (Figure 4, F–H and Supplemental Figure 5, H–J). We further found by limiting dilution assay that NRF2 KO1 or KO2 inhibited tumor initiation of MDA-MB-231 and MCF-7 in NSG mice (Figure 4I and Supplemental Figure 5K). Collectively, these results indicate that NRF2 is activated in BCSCs and phenocopies ZMYND8 for inhibition of ROS and ferroptosis and for maintenance of BCSCs in vitro and in vivo.

To determine whether ZMYND8 reduces ROS through activation of NRF2, we knocked out NRF2 in ZMYND8-OE MDA-MB-231 and MCF-7 cells (Figure 4F and Supplemental Figure 5H). ZMYND8 OE lost its ability to reduce ROS in NRF2 KO1 or KO2 mammospheres (Figure 4G and Supplemental Figure 5I). These results indicate that ZMYND8 increases ROS clearance in BCSCs in an NRF2-dependent manner.

### ZMYND8 interacts with NRF2 in BCSCs.

**Figure 5:**
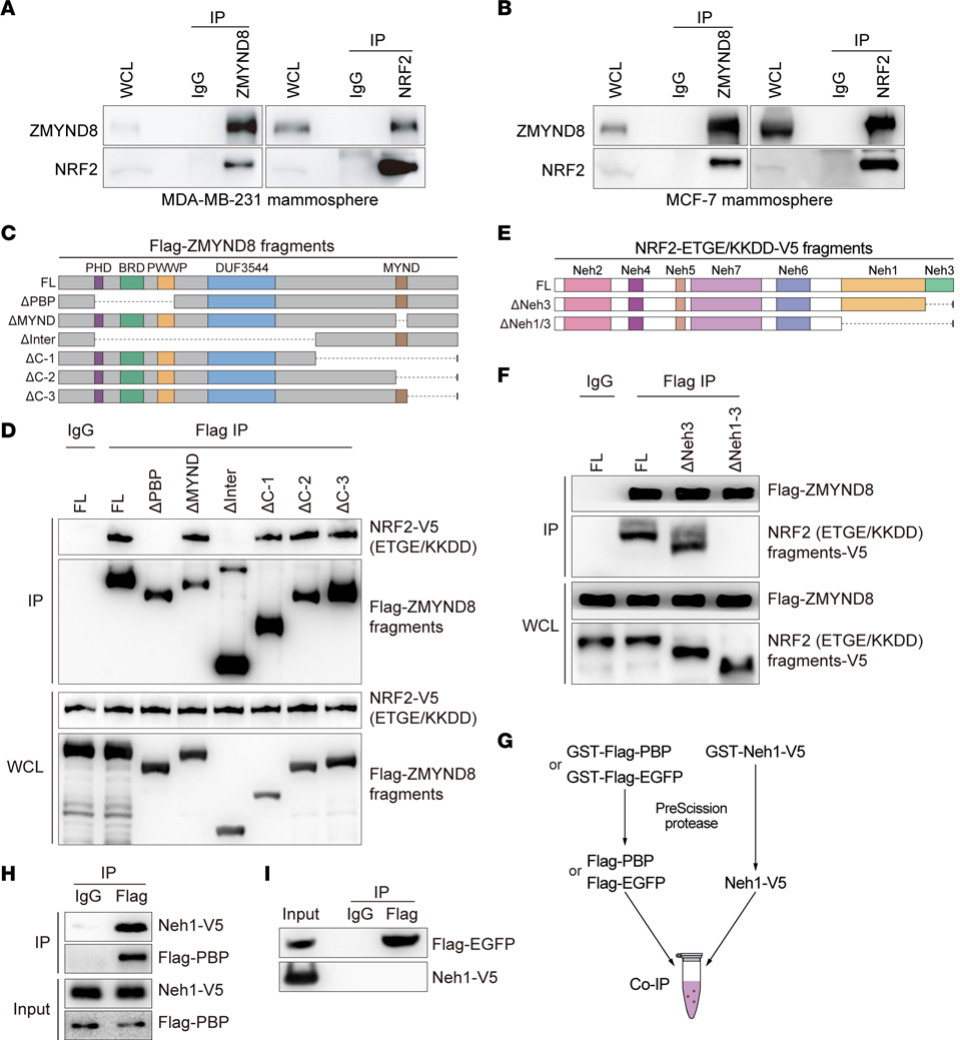
ZMYND8 directly interacts with NRF2. (**A** and **B**) Co-IP assay of endogenous ZMYND8 and NRF2 in MDA-MB-231 spheres (**A**) and MCF-7 spheres (**B**) (*n* = 3). (**C**) Schematic depiction of FL and truncated ZMYND8 proteins. (**D**) Co-IP assay of NRF2-ETGE/KKDD-V5 and FL or truncated Flag-ZMYND8 in transfected HEK293T cells (*n* = 3). (**E**) Schematic depiction of FL and truncated NRF2-ETGE/KKDD-V5. (**F**) Co-IP assay of Flag-ZMYND8 and FL or truncated NRF2-ETGE/KKDD-V5 in transfected HEK293T cells (*n* = 3). (**G**) Scheme of purification of Flag-PBP, Flag-EGFP, and Neh1-V5 proteins. (**H** and **I**) Co-IP assay of purified Flag-PBP (**H**) or Flag-EGFP (**I**) with Neh1-V5 (*n* = 3).

We next tried to dissect the mechanism by which ZMYND8 enhances NRF2 activation in BCSCs. To this end, we first assessed whether ZMYND8 interacts with NRF2 in BCSCs. Co-IP assay with anti-ZMYND8 antibody or control IgG showed that endogenous ZMYND8, but not IgG, pulled down endogenous NRF2 in MDA-MB-231 and MCF-7 mammospheres (Figure 5, A and B, left). A reciprocal co-IP assay with an anti-NRF2 antibody confirmed the physical interaction of NRF2 with ZMYND8 in MDA-MB-231 and MCF-7 mammospheres (Figure 5, A and B, right).

Next, we mapped the ZMYND8 domain binding to NRF2. HEK293T cells were cotransfected with vector encoding full-length (FL) or domain-deleted Flag-ZMYND8 (Figure 5C) and vector encoding FL NRF2-V5 with ETGE mutation into KKDD (NRF2-ETGE/KKDD-V5), which stabilizes NRF2 protein. Co-IP assay with anti-Flag antibody or IgG showed that MYND-deleted (ΔMYND), C-1-deleted (ΔC-1), C-2-deleted (ΔC-2), or C-3-deleted (ΔC-3) Flag-ZMYND8 interacted with NRF2-ETGE/KKDD-V5, similar to FL Flag-ZMYND8 (Figure 5D). In contrast, deletion of the PBP domain (ΔPBP) or interfragment (ΔInter) abolished ZMYND8 binding to NRF2 in HEK293T cells (Figure 5D). These results indicate that the PBP domain of ZMYND8 is required for interaction with NRF2.

Next, we mapped the NRF2 domain binding to ZMYND8. HEK293T cells were cotransfected with vector encoding FL, Neh3 domain-deleted (ΔNeh3), or Neh1 and Neh3 domains-deleted (ΔNeh1/3) NRF2-ETGE/KKDD-V5 (Figure 5E) and vector encoding FL Flag-ZMYND8. Co-IP assay with anti-Flag or IgG antibody showed that FL and ΔNeh3 similarly interacted with Flag-ZMYND8 (Figure 5F). However, ΔNeh1/3 abolished NRF2 binding to Flag-ZMYND8 (Figure 5F). These results indicate that the Neh1 domain of NRF2 is required for interaction with ZMYND8.

We next assessed whether ZMYND8 directly interacted with NRF2. GST-Flag-PBP, GST-Flag- EGFP, and GST-Neh1-V5 were expressed in bacteria, purified, and equally incubated with each other after GST removal (Figure 5G). Co-IP assay using anti-Flag antibody or control IgG showed robust interaction of Flag-PBP and Neh1-V5 (Figure 5H). However, Flag-EGFP had no interaction with Neh1-V5 (Figure 5I). These results indicate that the PBP domain of ZMYND8 directly interacts with the Neh1 domain of NRF2.

### ZMYND8 enhances the expression of NRF2-dependent antioxidant genes in BCSCs.

**Figure 6:**
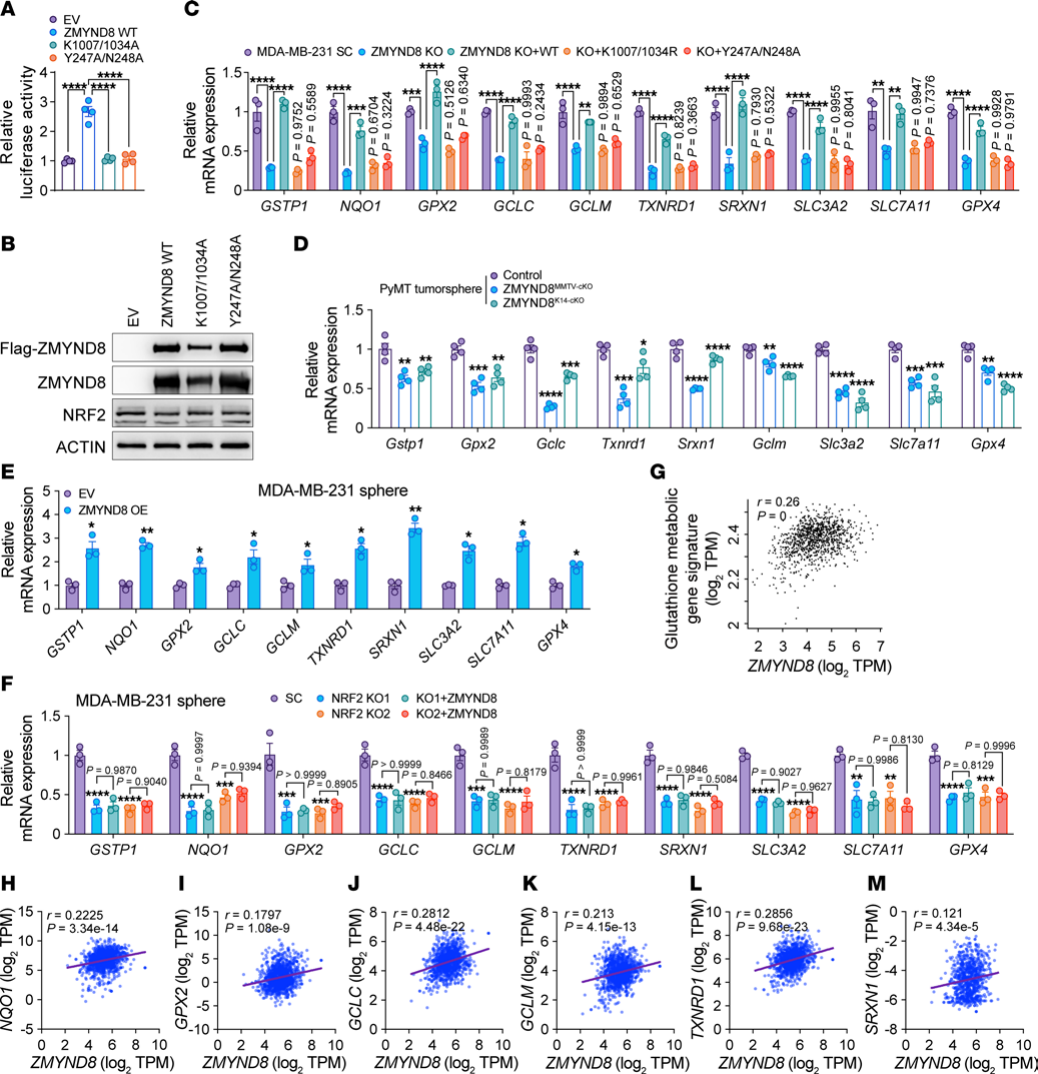
ZMYND8 enhances the transcription activity of NRF2. (**A** and **B**) NRF2 luciferase reporter assay in HEK293T cells cotransfected with indicated plasmids. The firefly/*Renilla* luciferase activity is quantified (**A**) (*n* = 4). Analysis of ZMYND8 and NRF2 protein levels (**B**). (**C** and **D**) mRNA analysis of antioxidant genes in MDA-MB-231-SC, ZMYND8 KO, and ZMYND8 KO rescued with WT ZMYND8, K1007/1034R, or Y247A/N248A mammospheres (**C**) (*n* = 3), control (*MMTV-PyMT*^–/+^ and *Zmynd8*^+/+^), ZMYND8^MMTV–cKO^ (*MMTV-PyMT*^–/+^, *MMTV-Cre*^–/+^, and *Zmynd8^fl/fl^*), ZMYND8^K14–cKO^ (*MMTV-PyMT*^–/+^, *K14-Cre*^–/+^, and *Zmynd8^fl/fl^*) tumorspheres (**D)** (*n* = 4). (**E**) mRNA analysis of antioxidant genes in MDA-MB-231 mammospheres expressing EV or ZMYND8 (*n* = 3). (**F**) mRNA analysis of antioxidant genes in MDA-MB-231-SC, NRF2 KO1/2, and NRF2 KO1/2 plus WT ZMYND8 mammospheres (*n* = 3). (**G**) Correlation analysis between *ZMYND8* mRNA and glutathione metabolic gene signature in human breast tumors from TCGA cohort. Data were retrieved from GEPIA2. (**H**–**M**) Correlation analysis between *ZMYND8* and antioxidant gene mRNAs in human breast tumors from TCGA cohort. Data represent mean ± SEM. *P* values were determined by using 1-way ANOVA corrected with Tukey’s test (**A**, **C**, and **F**) or Dunnett’s test (**D**), 2-tailed Student’s *t* test (**E**). *P < 0.05; **P < 0.01; ***P < 0.001; ****P < 0.0001.

To determine whether ZMYND8 is required for NRF2-mediated transactivation, we first performed NRF2-dependent luciferase reporter assay. HEK293T cells were transfected with NRF2 luciferase reporter plasmid, control plasmid pSV-*Renilla*, and expression vector encoding WT, K1007/1034R, or Y247A/N248A Flag-ZMYND8, or empty vector (EV). Expression of WT Flag-ZMYND8 significantly increased NRF2 transcriptional activity in HEK293T cells, whereas K1007/1034R or Y247A/N248A Flag-ZMYND8 had no effect (Figure 6A). NRF2 protein expression was not affected by Flag-ZMYND8 in HEK293T cells (Figure 6B). We next assessed the effect of ZMYND8 on mRNA expression of NRF2 target genes. ZMYND8 KO significantly decreased mRNA expression of *GSTP1*, *NQO1*, *GPX2*, *GCLC*, *GCLM*, *TXNRD1*, *SRXN1*, *SLC3A2*, *SLC7A11*, and *GPX4* in MDA-MB-231 and MCF-7 mammospheres, which was restored by reexpression of WT ZMYND8 (Figure 6C and Supplemental Figure 6A). Neither K1007/1034R nor Y247A/N238A mutant had a rescued effect in MDA-MB-231 mammospheres (Figure 6C). Consistently, 0.5 μM JQ1 treatment mimicked ZMYND8 KO to suppress these NRF2 target genes (Supplemental Figure 6B). Likewise, mRNA expression of *Gstp1*, *Gpx2*, *Gclc*, *Txnrd1*, *Srxn1*, *Gclm*, *Slc3a2*, *Slc7a11*, and *Gpx4* was also decreased in ZMYND8^MMTV–cKO^ and ZMYND8^K14–cKO^ tumorspheres ex vivo compared with control tumorspheres (Figure 6D). In contrast, ZMYND8 OE significantly increased mRNA expression of *GSTP1*, *NQO1*, *GPX2*, *GCLC*, *GCLM*, *TXNRD1*, *SRXN1*, *SLC3A2*, *SLC7A11*, and *GPX4* in both MDA-MB-231 and MCF-7 mammospheres (Figure 6E and Supplemental Figure 6C), but failed to do so in NRF2 KO1 or KO2 mammospheres (Figure 6F and Supplemental Figure 6D). These results indicate that ZMYND8 enhanced the transcription of antioxidant genes reliant on NRF2. To further determine specific regulation of NRF2 activation by ZMYND8, we overexpressed Flag-NRF2-ETGE/KKDD in ZMYND8 KO MDA-MB-231 mammospheres and found that NRF2 OE failed to activate 9 out of 10 antioxidant genes we tested in ZMYND8 KO mammospheres (Supplemental Figure 6, E and F). Likewise, *NQO1*, but not other antioxidant genes, was fully restored by NRF2 OE in both ZMYND8 KO1 and KO2 MCF-7 mammospheres (Supplemental Figure 6, G and H), suggesting that ZMYND8 was necessary for the transcription of most NRF2 target genes, though it was not the exclusive coactivator for the NRF2 target gene *NQO1*. Lastly, we performed gene correlation analysis in human breast tumors to support positive regulation of NRF2 target genes by ZMYND8. Gene expression analysis in the Cancer Genome Atlas (TCGA) cohort revealed that *ZMYND8* mRNA significantly correlated with the gene signature of glutathione metabolic process in human breast tumors (Figure 6G). Pair-wise mRNA expression correlation analysis in human breast tumors from TCGA cohort showed a positive correlation between *ZMYND8* and antioxidant genes including *NQO1*, *GPX2*, *GCLC*, *GCLM*, *TXNRD1*, and *SRXN1* (Figure 6, H–M). Taken together, these results indicate that ZMYND8 enhanced the transcriptional activity of NRF2 through its epigenetic activities leading to transcription of antioxidant genes in BCSCs.

### ZMYND8 recruits NRF2 to the promoters of NRF2-dependent antioxidant genes.

**Figure 7:**
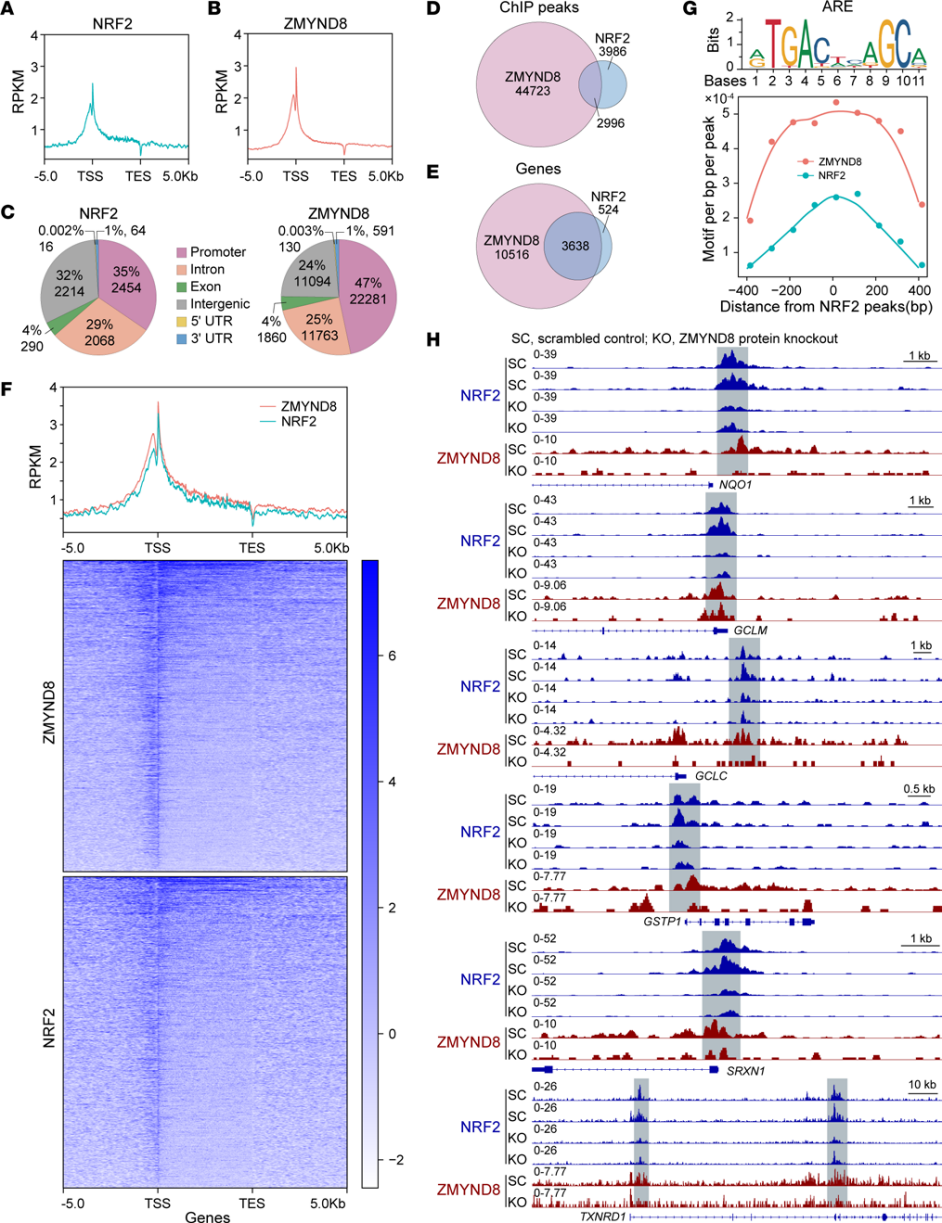
ZMYND8 recruits NRF2 to the promoters of antioxidant genes. (**A-C**) Metagene analysis of the genomic distribution of NRF2 (**A** and **C**) and ZMYND8 (**B** and **C**) in MDA-MB-231 mammospheres (*n* = 2). RPKM, reads per kilobase of transcript per million reads mapped; TSS, transcription start site; TES, transcription end site. (**D** and **E**) Venn diagram of the overlapped ChIP-Seq peaks (**D**) and cooccupied genes (**E**) by ZMYND8 and NRF2 (*n* = 2). (**F**) Cooccupancy analysis of NRF2 and ZMYND8 ChIP-Seq peaks (*n* = 2). (**G**) Motif density analysis of ZMYND8 ChIP-Seq peaks (*n* = 2). ARE is shown in the top panel. (**H**) Genome browser snapshots of NRF2 and ZMYND8 ChIP-Seq peaks on antioxidant genes.

To determine whether ZMYND8 directly coactivates NRF2-dependent antioxidant genes, we performed ChIP-Seq in SC and ZMYND8 KO MDA-MB-231 mammospheres. Both ZMYND8 and NRF2 were highly enriched near the transcription start site in SC mammospheres (Figure 7, A and B). Their ChIP-Seq peaks were mainly located at the promoter (47% of ZMYND8 and 35% of NRF2 peaks), intron (25% of ZMYND8 and 29% of NRF2 peaks), and intergenic region (24% of ZMYND8 and 32% of NRF2 peaks) (Figure 7C). Remarkably, 42.9% of NRF2 ChIP-Seq peaks overlapped with ZMYND8 ChIP-Seq peaks (Figure 7D), and 87.4% of NRF2 target genes were cooccupied by NRF2 and ZMYND8 (Figure 7E). The metagene analysis confirmed strong colocalization of NRF2 and ZMYND8 (Figure 7F). The motif analysis of ZMYND8 ChIP-Seq peaks revealed marked enrichment of the ARE motif (Figure 7G). These data indicate genome-wide colocalization of NRF2 and ZMYND8 at the AREs in BCSCs.

We next assessed whether ZMYND8 controls NRF2 binding to antioxidant genes. ZMYND8 KO remarkably decreased enrichment of NRF2 and ZMYND8 on *NQO1*, *GCLM*, *GCLC*, *GSTP1*, *SRXN1*, and *TXNRD1* genes in MDA-MB-231 mammospheres (Figure 7H). ZMYND8 is known to recognize H3K14ac (23). We found cooccupation of ZMYND8 and H3K14ac on these antioxidant genes in MDA-MB-231 cells (Supplemental Figure 7). Collectively, these results indicate that ZMYND8 recruits NRF2 to the AREs on antioxidant genes leading to gene transcription in BCSCs.

### ZMYND8 increases NRF2 protein stability through KEAP1 repression.

**Figure 8:**
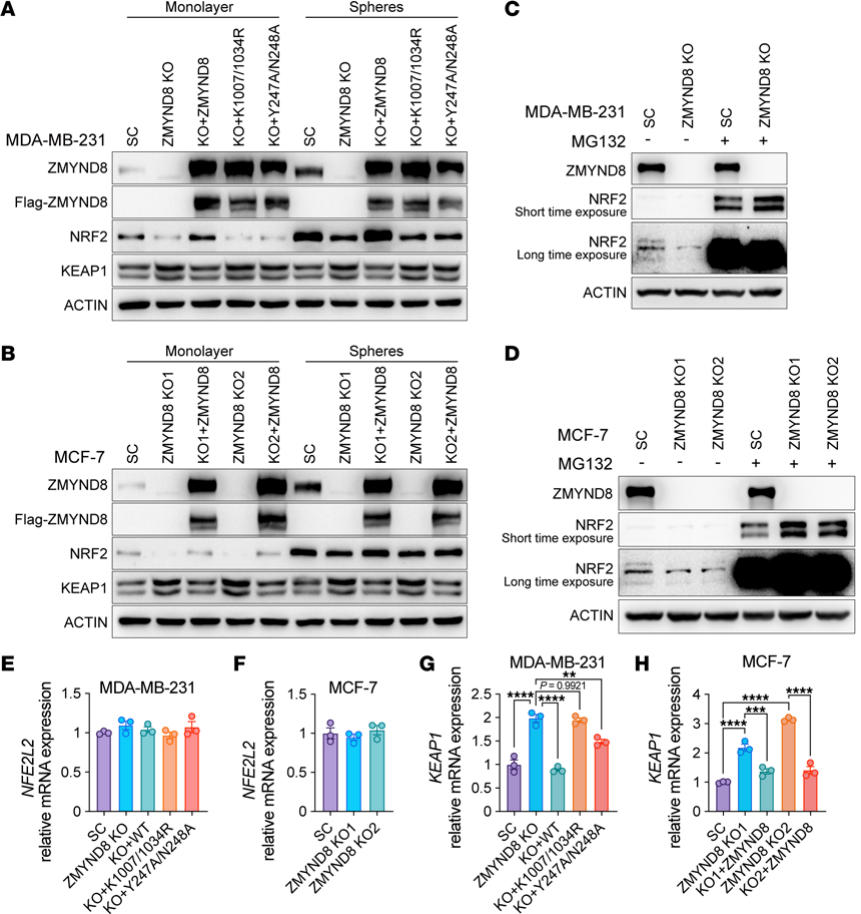
ZMYND8 increases NRF2 protein stability through KEAP1 silencing. (**A**) Analysis of ZMYND8, KEAP1, and NRF2 protein levels in MDA-MB-231-SC, ZMYND8 KO, and ZMYND8 KO rescued with WT ZMYND8, K1007/1034R, or Y247A/N248A monolayers and mammospheres (*n* = 3). (**B**) Analysis of ZMYND8, KEAP1, and NRF2 protein levels in MCF-7-SC, ZMYND8 KO1/2, and ZMYND8 KO1/2 rescued with WT ZMYND8 monolayers and mammospheres (*n* = 3). (**C**) Analysis of NRF2 and ZMYND8 protein levels in MDA-MB-231-SC and ZMYND8 KO cells treated with DMSO (–) or MG132 (+, 10 μM for 2 hrs and 2.5 μM for an additional 22 hrs). *n* = 3. (**D**) Analysis of NRF2 and ZMYND8 protein levels in MCF-7-SC and ZMYND8 KO1/2 cells treated with DMSO (-) or MG132 (+). *n* = 3. (**E** and **G**) Analysis of *NFE2L2* (**E**) and *KEAP1* (**G**) mRNAs in MDA-MB-231-SC, ZMYND8 KO, and ZMYND8 KO rescued with WT ZMYND8, K1007/1034R, or Y247A/N248A mammospheres (*n* = 3). (**F**) Analysis of *NFE2L2* mRNA in MCF-7-SC and ZMYND8 KO1/2 mammospheres (*n* = 3). (**H**) Analysis of *KEAP1* mRNA in MCF-7-SC, ZMYND8 KO1/2, and ZMYND8 KO1/2 rescued with WT ZMYND8 mammospheres (*n* = 3). Data represent mean ± SEM. *P* value was determined by using 1-way ANOVA corrected with Tukey’s test (**E**, **G**, and **H**) or Dunnett’s test (**F**). **P < 0.01; ***P < 0.001; ****P < 0.0001.

We found that ZMYND8 KO reduced NRF2 protein levels in MDA-MB-231 monolayers and mammospheres, which was restored by reexpression of WT ZMYND8 but not K1007/1034R or Y247A/N238A mutant (Figure 8A). However, the inhibition of NRF2 protein by ZMYND8 KO was minimal in MCF-7 cells, especially in mammospheres (Figure 8B). Treatment with proteasome inhibitor MG132 blocked NRF2 protein reduction in ZMYND8-KO MDA-MB-231 and MCF-7 monolayers (Figure 8, C and D). *NFE2L2* mRNA levels were not controlled by ZMYND8 in MDA-MB-231 and MCF-7 mammospheres (Figure 8, E and F). These results suggest that ZMYND8 increased NRF2 protein stability in certain breast cancer cells, which was not BCSCs-specific.

We next studied whether ZMYND8 controlled NRF2 protein stability through KEAP1. KEAP1 protein was induced by ZMYND8 KO, which was reversed by reexpression of WT ZMYND8 in MDA-MB-231 and MCF-7 monolayers and mammospheres (Figure 8, A and B). Neither K1007/1034R nor Y247A/N238A mutant had an effect on KEAP1 protein levels (Figure 8A). The negative regulation of KEAP1 by ZMYND8 was also observed at the mRNA level in MDA-MB-231 and MCF-7 mammospheres (Figure 8, G and H). Taken together, these results suggest that ZMYND8 suppressed *KEAP1* transcription through its epigenetic activities leading to increased NRF2 protein stability.

### NRF2 activates ZMYND8 transcription through binding the ARE at the ZMYND8 promoter.

**Figure 9:**
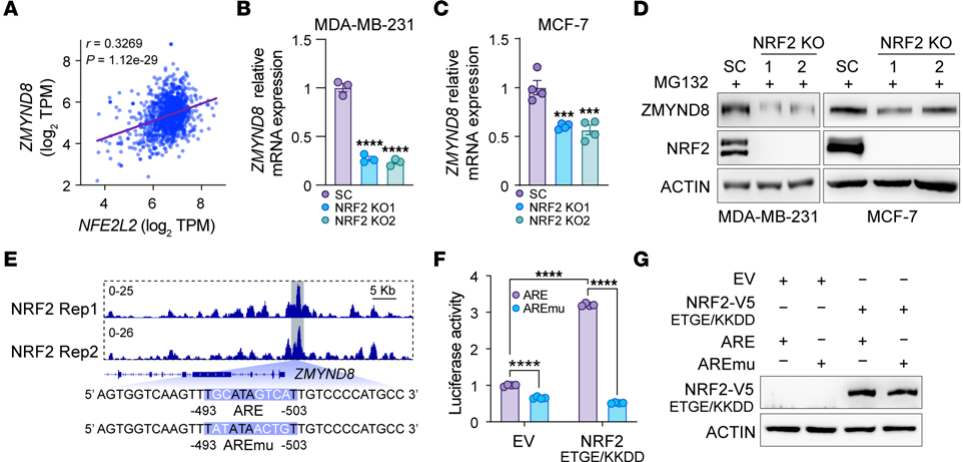
*ZMYND8* is a direct NRF2 target gene. (**A**) Correlation analysis between *ZMYND8* and *NFE2L2* mRNAs in human breast tumors from TCGA cohort. (**B** and **C**) Analysis of *ZMYND8* mRNA in MDA-MB-231-SC, (**B**) and MCF-7-SC, (**C**) NRF2 KO1, and NRF2 KO2 mammospheres (*n* = 3–4). (**D**) Analysis of ZMYND8 and NRF2 protein levels in MDA-MB-231-SC and MCF-7-SC, NRF2 KO1, and NRF2 KO2 cells after MG132 (10 μM) treatment (*n* = 3). (**E**) NRF2 enrichment at the *ZMYND8* promoter in MDA-MB-231 mammospheres (top). The *ZMYND8* ARE sequence is shown at the bottom. Rep, replicate; AREmu, ARE mutant. (**F** and **G**) Luciferase reporter assay in HEK293T cells cotransfected with indicated plasmids. The firefly/*Renilla* luciferase activity is quantified (**F**) (*n* = 4). Analysis of NRF2 protein levels (**G**). Data represent mean ± SEM. *P* value was determined by using 1-way ANOVA corrected with Dunnett’s test (**B** and **C**) and 2-way ANOVA corrected with Tukey’s test (**F**). ***P < 0.001; ****P < 0.0001.

We found a positive correlation between *ZMYND8* and *NFE2L2* mRNAs in human breast tumors and 28 other types of human cancers from TCGA cohort (Figure 9A and Supplemental Figure 8). Interestingly, mRNA expression of *ZMYND8* was reduced in NRF2 KO1 and KO2 MDA-MB-231 or MCF-7 mammospheres compared with SC mammospheres (Figure 9, B and C). Consistently, NRF2 KO decreased ZMYND8 protein expression in MDA-MB-231 and MCF-7 mammospheres (Figure 9D). These results indicate that NRF2 was an upstream regulator of ZMYND8.

To determine whether *ZMYND8* was a direct NRF2 target gene, we analyzed NRF2 ChIP-Seq data in MDA-MB-231 mammospheres and found a robust binding peak of NRF2 at the promoter of *ZMYND8* gene (Figure 9E). A putative ARE was identified within the NRF2 binding region (Figure 9E). To determine whether NRF2 activated *ZMYND8* transcription through this ARE, we cloned the DNA sequence flanking the *ZMYND8* ARE (Figure 9E) into a firefly luciferase reporter vector pGL2. A mutant *ZMYND8* ARE (AREmu; Figure 9E) plasmid was similarly generated as a negative control. HEK293T cells were cotransfected with vector encoding NRF2-ETGE/KKDD-V5 or EV, pSV-*Renilla*, and WT or mutant *ZMYND8* ARE luciferase reporter vector. Compared with EV, NRF2 OE significantly increased the WT *ZMYND8* ARE reporter activity but had no effect on the mutant *ZMYND8* ARE reporter activity (Figure 9F). Protein expression of NRF2-ETGE/KKDD-V5 was comparable when coexpressed with WT or mutant *ZMYND8* ARE reporter (Figure 9G). Collectively, these results support that NRF2 directly activated *ZMYND8* transcription through binding the ARE at the *ZMYND8* promoter.

## Discussion

**Figure 10:**
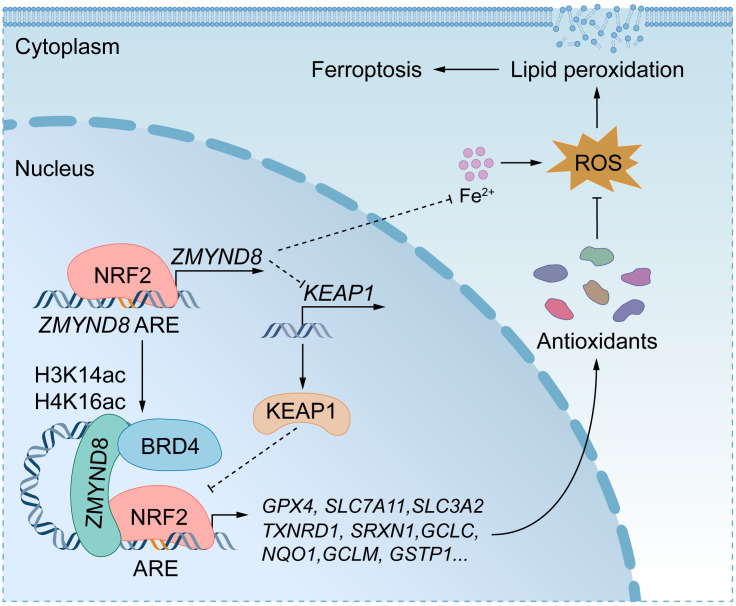
A proposed model for ZMYND8-induced antioxidant defense mechanism protecting BCSCs against ferroptosis. ZMYND8 increases NRF2 protein stability through KEAP1 silencing and enhances recruitment of NRF2 to antioxidant genes in BCSCs, leading to their gene transcription. Additionally, ZMYND8 reduces iron levels in BCSCs. Collectively, ZMYND8 effectively blocks ferroptosis in BCSCs. ZMYND8 is induced by NRF2, which provides a feedback loop mitigating oxidative stress and ferroptosis in BCSCs to promote BCSC expansion and tumor initiation.

In the present study, we identified an epigenetic mechanism underlying the fate of BCSCs. ZMYND8 and NRF2 are induced in ALDH^hi^ BCSCs and cooperate to inhibit BCSC ferroptosis leading to tumor initiation in mice. *ZMYND8* is a direct NRF2 target gene, and it therefore provides a positive feedback mechanism that amplifies NRF2 activation to protect ALDH^hi^ BCSCs against oxidative stress and ferroptosis (Figure 10). This feedback mechanism might be expanded to 28 other types of human cancers because of a significant correlation between *ZMYND8* and *NFE2L2* mRNAs in these cancers.

BCSCs exhibit resistance to various treatment modalities. Although the regulatory mechanism governing the fate of BCSCs is complex, emerging studies have shown that BCSCs are highly susceptible to ferroptosis because high concentrations of iron, an essential element of ferroptosis, are found in BCSCs (5). Interestingly, ZMYND8 decreases iron levels in BCSCs. A recent report revealed a similar role of NRF2 in iron homeostasis (24), suggesting that ZMYND8 may coordinate with NRF2 to regulate iron homeostasis in BCSCs. Additionally, ZMYND8 promotes ROS clearance in BCSCs. Thus, ZMYND8 attenuates 2 key elements to inhibit ferroptosis in BCSCs, which represents an epigenetic mechanism of ferroptosis. While oxidative stress was shown to promote the transition of mesenchymal-BCSCs (CD44^+^CD24^–^) to epithelial-BCSCs (ALDH^hi^) states (25), ZMYND8 unlikely controls this transition through ROS because it has been shown to promote expansion of both CD44^+^CD24^–^ and ALDH^hi^ BCSCs (12). Collectively, the oncogene ZMYND8 has a multifaceted role in regulation of BCSC fate.

Although it is expressed ubiquitously in all cell types, NRF2 is rarely detected at the protein level under normal conditions due to KEAP1-mediated proteasomal degradation (10, 11). Genetic mutations in *NFE2L2* and *KEAP1* are uncommon in human breast tumors (10). We showed that ZMYND8 is a critical regulator of NRF2 protein stability through suppressing KEAP1, although NRF2 protein stability is modestly regulated by ZMYND8 in MCF-7 cells. It is noteworthy that KEAP1 protein levels are not altered in BCSCs compared with non-BCSCs, suggesting that NRF2 protein induction is KEAP1-independent in BCSCs. Together, our findings implicate that NRF2 protein stability partially contributes to ZMYND8-induced antioxidant genes.

We showed that ZMYND8 enhanced the transcription of antioxidant genes by recruiting NRF2, suggesting that ZMYND8 acts as a coactivator of NRF2 in BCSCs. The modest gene correlations between ZMYND8 and glutathione metabolic gene signature or antioxidant genes in TCGA breast tumors are likely due to limitations of analysis of overall gene expression in the mixed cell populations. We demonstrated that the reader cassette PBP of ZMYND8 directly interacts with the Neh1 domain of NRF2, where multiple lysine residues are acetylated by histone acetyltransferases p300/CBP and MOF and arginine 437 residue is methylated by arginine methyltransferase 1 (26–28). The Neh1 domain is responsible for NRF2 binding to the ARE and its acetylation or methylation promotes NRF2-DNA binding and the subsequent antioxidant gene expression (29). Our previous study showed that ZMYND8 physically interacts with p300 in breast cancer cells (16). Whether or not ZMYND8 recognizes acetylated and/or methylated NRF2 to enhance its transcriptional activity in BCSCs requires further investigation in the future. Nevertheless, our findings indicate that ZMYND8 may recruit BRD4 to enhance the transcriptional activity of NRF2, during which process K1007 and K1034 acetylation of ZMYND8 is crucial (16). Together, our current work unveils a molecular mechanism involving a two-fold posttranslational regulation of NRF2 mediated by a histone reader protein.

NRF2 has been shown to play a complex role in tumor initiation and progression. NRF2 prevents accumulation of damaged cellular components caused by oxidative stress in normal cells, thus inhibiting tumor initiation in carcinogen-induced mouse cancer models (11, 30). However, in established tumors, hyperactivation of NRF2 promotes tumor progression by protecting cancer cells against excessive oxidative stress (11, 31, 32). BCSCs often reside at the hypoxic tumor microenvironment, where elevated levels of oxidative stress are detected (33). Our data here uncover that activation of NRF2 by ZMYND8 triggers an active defense system allowing BCSCs to better cope with potential oxidative stress and ferroptosis for survival and stemness, suggesting that the role of NRF2 is highly dependent on the cellular and environmental context in human cancers. Hyperactivation of NRF2 is also associated with several types of therapy resistance in cancer, including chemoresistance, radioresistance, targeted therapy resistance, and immunotherapy resistance (10, 34–36). Given that ZMYND8 has been shown to promote radioresistance in glioma (37), it would be interesting to study whether ZMYND8-mediated NRF2 activation in BCSCs could promote resistance to therapies in breast cancer.

In conclusion, ZMYND8 acted as an epigenetic regulator of NRF2 to enhance the expression of antioxidant genes and reduced iron levels in BCSCs, which protected BCSCs against ferroptosis leading to tumor initiation (Figure 10). Thus, we believe that ZMYND8 would be an effective therapeutic target for the treatment of breast cancer and other human cancers with high activation of NRF2.

## Methods

### Sex as a biological variable.

Our study exclusively examined female mice because we studied breast cancer in women.

### Animal studies.

*Zmynd8*^MMTV–cKO^ and *Zmynd8*^K14–cKO^ mice were generated and crossed with MMTV-PyMT transgenic mice as described previously (12). For limiting dilution assay, MDA-MB-231, MCF-7, or 4T1 cells suspended in 100 μL of PBS/Matrigel (1:1, Corning) were implanted into the 2nd left mammary fat pad of 6-to-8 week-old female NSG (the Jackson Laboratory, no. 005557) or BALB/c (the Jackson Laboratory, no.000651) mice. Tumor initiation was confirmed after mouse euthanization at postimplantation day 45 for MDA-MB-231 and MCF-7 cells, or at postimplantation day 30 for 4T1 cells. For tumor growth, 0.1 million 4T1 cells were implanted into the 2nd left mammary fat pad of female NSG or BALB/c mice. 4T1 tumors were measured starting from day 8 and harvested at day 21 after implantation. A slow-release 17-β-estradiol pellet (0.72 mg/60-day release/pellet, Innovative Research of America) was implanted subcutaneously into mice the day before MCF-7 cell implantation. For NAC treatment, mice were administrated for 45 days with NAC (1 g/L, Sigma-Aldrich) in drinking water.

### Cell culture and lentivirus.

MDA-MB-231 (a gift from R. Brekken, UT Southwestern, Dallas, TX, USA), MCF-7 (American Type Culture Collection), and human embryonic kidney (HEK) 293T and HEK293FT (Invitrogen) cells were cultured in DMEM (Sigma-Aldrich) supplemented with 10% heat-inactivated FBS (Sigma-Aldrich) at 37°C in a 5% CO_2_/95% air incubator. 4T1 cells (a gift from Y. Fu, UT Southwestern) were cultured in RPMI-1640 medium (Sigma-Aldrich) supplemented with 10% FBS at 37°C in a 5% CO_2_/95% air incubator. CRISPR/Cas9 sgRNAs for NRF2 KO were listed in Supplemental Table 1, the other KO cell lines have been described previously (12, 16). Lentivirus was generated in HEK293FT cells as described previously (12, 16). All cell lines were mycoplasma-free and authenticated by short tandem repeat DNA profiling analysis.

### Mammosphere formation and self-renewal assays.

MDA-MB-231 or MCF-7 cells were trypsinized to single-cell suspensions, washed with HBSS, resuspended in MammoCult medium (STEMCELL Technologies) with or without DMSO (Sigma-Aldrich), 1 mM NAC (Sigma-Aldrich), 1 μM GSH-EE (Sigma-Aldrich), 2.5 μM Ferrostatin-1 (Thermo Fisher Scientific), 20 μM Z-VAD-FMK (Thermo Fisher Scientific), 10 μM Olaparib (Thermo Fisher Scientific), 0.5 μM JQ1 (MedChemExpress), 10 μM Erastin (MedChemExpress), or 1 μM Liproxstatin-1 (MedChemExpress), and cultured for 4-to-7 days on an ultralow attachment 6-well plate at 37°C in a 5% CO_2_/95% air incubator. Mammospheres were imaged under a Zeiss Axio Observer Z1 microscope.

For the self-renewal assay, all primary mammospheres were harvested and trypsinized to single-cell suspensions, then seeded on ultralow attachment 6-well plates for secondary mammosphere culture. The fold change of expansion and self-renewal was calculated as follows: (secondary − primary mammosphere number)/primary mammosphere number.

PyMT tumor spheres were generated as described previously (12). Briefly, the tumors were harvested, crosscut, washed, and digested for 45 minutes with gentle collagenase/hyaluronidase (STEMCELL Technologies) in a 37°C shaker. After filtering with a 40-μm cell strainer (Thermo Fisher Scientific) and centrifugation, single cells were resuspended in DMEM/Ham’s F-12 medium with B-27 supplement (Thermo Fisher Scientific), EGF (20 ng/mL, Sigma-Aldrich), basic fibroblast growth factor (20 ng/mL, STEMCELL Technologies), heparin (4 μg/mL, STEMCELL Technologies), and 1% penicillin/streptomycin/neomycin (Sigma-Aldrich) and plated overnight on a collagen I–coated plate (Thermo Fisher Scientific) at 37°C in a 5% CO_2_/95% air incubator. The next day, cells were trypsinized and reseeded on an ultralow attachment dish (Corning) at 37°C in a 5% CO_2_/95% air incubator for continuous incubation for 7 days. Tumor spheres were harvested for protein and mRNA analyses.

### Cell death assay.

Monolayer cells or 4-day-cultured mammospheres were trypsinized and dissociated into single-cell suspensions. ALDH^hi^ BCSCs were isolated with the ALDEFLUOR kit according to the manufacturer’s protocol (STEMCELL Technologies, no. 01700), followed by staining with PI (1 μg/mL, Sigma-Aldrich) for 15 minutes at 25°C. The percentage of PI-positive BCSCs was examined on a FACSCanto RUO cell analyzer (Becton Dickinson).

### Cellular ROS assay.

Cellular ROS in spheres was measured with a cellular ROS assay kit (Abcam, no. ab113851) according to the manufacturer’s protocol. The fluorescence of 2′,7′-dichlorofluorescein was normalized to cell number. Cellular ROS in ALDH^hi^ BCSCs and ALDH^lo^ non-BCSCs was measured with a CellROX deep red flow cytometry assay kit (Invitrogen, no. C10491) according to the manufacturer’s protocol. Cells were examined on a FACSCanto RUO cell analyzer (Becton Dickinson) or Cytek Northern Lights flow cytometer (Cytek Biosciences).

### GSH/GSSG ratio assay.

Mammospheres were trypsinized and dissociated into single-cell suspensions after 4-day cultures. Cellular GSH and GSSG levels were measured with a GSH/GSSG ratio detection assay kit (Abcam, no. ab205811) according to the manufacturer’s protocol.

### Lipid peroxidation assay.

Mammospheres were trypsinized and dissociated into single-cell suspensions after 4-day cultures. Lipid peroxidation assay was performed with a lipid peroxidation assay kit (Abcam, no. ab118970) according to the manufacturer’s protocol.

### Iron assay.

Mammospheres were trypsinized and dissociated into single-cell suspensions after 4-day cultures. Cellular Fe^2+^ was measured with an iron assay kit (Sigma-Aldrich, no. MAK025) according to the manufacturer’s protocol.

### Cell sorting.

Cells were trypsinized, dissociated into single-cell suspensions, and stained with the ALDEFLUOR kit according to the manufacturer’s protocol (STEMCELL Technologies). ALDH^hi^ BCSCs and ALDH^lo^ non-BCSCs were sorted on a FACSAria II SORP cell sorter (Becton Dickinson).

### Plasmid constructs.

FL *NFE2L2* cDNA was cloned into pcDNA3.1-V5/His-B vector, and NRF2-ETGE/KKDD mutant was generated by site-directed mutagenesis PCR. NRF2-ETGE/KKDD cDNA was subcloned to pLVX-UbC-Flag-N vector. ZMYND8 PBP domain or FL EGFP fused with N-terminal 3×Flag or NRF2 Neh1 domain fused with C-terminal V5 was cloned into pGEX-6P-1 vector. WT or mutant *ZMYND8* ARE with the flanking region was cloned into the pGL2-promoter vector. Other plasmids have been described previously (12, 16). All plasmids were confirmed by nucleotide sequence analysis.

### Co-IP and immunoblot assays.

Homogenized cells were lysed in NETN lysis buffer (150 mM NaCl, 1 mM EDTA, 10 mM Tris-HCl, pH 8.0, 0.5% Igepal, and protease inhibitor cocktail) for 30 minutes on ice, followed by sonication for 15 seconds. The supernatant was collected for IP or immunoblot after centrifugation. Protein concentration was determined with Bradford assay (Bio-Rad). For co-IP, the supernatant was incubated overnight with anti-ZMYND8 antibody (Bethyl Laboratories, no. A302-089A, RRID: 1604282), anti-NRF2 antibody (Proteintech, no. 66504-1-Ig, RRID: 2881867), anti-Flag antibody (Sigma-Aldrich, no. F3165, RRID: 259529), or IgG (Santa Cruz Biotechnology, no. sc-2025, RRID: 737182 or Cell Signaling Technology, no. 2729, RRID: 1031062) in the presence of protein A/G magnetic beads (Bio-Rad) at 4°C. After washing 4 times with NETN lysis buffer, the bound proteins were boiled in 1× Laemmli buffer for 5 minutes and fractionated by SDS-PAGE, followed by immunoblot assay with anti-V5 (Proteintech, no. 14440-1-AP, RRID: 2878059), anti-Flag (Sigma-Aldrich, no. F3165, RRID: 259529), anti-ZMYND8 (Bethyl Laboratories, no. A302-089A, RRID: 1604282), anti-NRF2 (Proteintech, no. 66504-1-Ig, RRID: 2881867), anti-KEAP1 (Santa Cruz Biotechnology, no. sc-365626, RRID: 10844829), or anti-actin (Sigma-Aldrich, no. A2066, RRID: 476693) antibody.

pGEX-6P-1-3×Flag-PBP, pGEX-6P-1-3×Flag-EGFP, or pGEX-6P-1-Neh1-V5 vector was transformed into *E*. *coli* BL21(DE3). After treatment with 1 mM IPTG for 4 hours at 25°C, bacteria were lysed in lysis buffer (50 mM Tris/HCl, pH 8.0, 150 mM NaCl, 10% glycerol, 0.1% Triton X-100, 0.1 mg/mL lysozyme, and protease inhibitor cocktail), followed by sonication for 3 minutes. After centrifugation, the supernatant containing GST-3×Flag-PBP, GST-3×Flag-EGFP, or GST-Neh1-V5 protein was purified with Glutathione Sepharose beads (Cytiva). The GST tag was cleaved away from these fusion proteins with PreScission protease (Cytiva). 3×Flag-PBP or 3×Flag-EGFP protein was mixed with Neh1-V5 protein in a 1:1 ratio for co-IP with anti-Flag antibody (Sigma-Aldrich, no. F3165, RRID: 259529) or IgG (Santa Cruz Biotechnology, no. sc-2025, RRID: 737182), followed by immunoblot assay with anti-V5 (Proteintech, no. 14440-1-AP, RRID: 2878059) or anti-Flag (Sigma-Aldrich, no. F3165, RRID: 259529) antibody.

### Luciferase reporter assay.

HEK293T cells were seeded onto a 48-well plate and transfected with pLenti-ARE-HSVTK-luc reporter plasmid (Addgene no. 161789), pGL2-promoter-*ZMYND8* WT ARE reporter plasmid, or pGL2-promoter-*ZMYND8* mutant ARE reporter plasmid; control reporter plasmid pSV-*Renilla*; and expression vector encoding ZMYND8, ZMYND8-K1007/1034A, ZMYND8-Y247A/N248A, NRF2-ETGE/KKDD, or EV. After 48 hours, the firefly and *Renilla* luciferase activities were measured by the Dual-Luciferase Assay System (Promega).

### Quantitative reverse-transcription PCR assay.

Total RNA was extracted from cultured cells with TRIzol reagent (Thermo Fisher Scientific), treated with DNase I (Ambion), and reverse transcribed using iScript reverse transcription supermix (Bio-Rad). qPCR was performed using iTaq universal SYBR green supermix (Bio-Rad) with primers listed in Supplemental Table 2. The mRNA fold change was calculated based on the threshold cycle (Ct) as 2 ^−Δ(ΔCt)^, where ΔCt = Ct_target_ − Ct_18S__rRNA_ and Δ(ΔCt) = ΔCt_KO_ _or_ _rescue_ − ΔCt_EV,_ _SC,_ _or_ _control_. The mRNA fold change was converted into a Z-score in the heatmap.

### ChIP-Seq assay.

Cells were cross-linked with 1% formaldehyde and quenched in 125 mM glycine. Chromatin was isolated using a SimpleChIP enzymatic chromatin IP kit (Cell Signaling Technology), sonicated to 200 to 300 bp in length, and subjected to IP with anti-ZMYND8 (Bethyl Laboratories, no. A302-089A, RRID: 1604282), anti-NRF2 (Cell Signaling Technology, no. 12721, RRID: 2715528), or anti-H3K14ac (Abcam, no. ab52946, RRID: 880442) antibody. ChIP DNA libraries were prepared with the NEBNext Ultra II DNA library prep kit for Illumina and sequenced on the Illumina NextSeq 2K. Bioinformatics were performed as described previously (16).

### TCGA data analysis.

RNA-Seq data from TCGA human cancer cohorts were downloaded from the UCSC Cancer Browser (38). *ZMYND8*, *NFE2L2*, *NQO1*, *GPX2*, *GCLC*, *GCLM*, *TXNRD1*, and *SRXN1* mRNA expression were queried in breast and other tumors, and pair-wise gene expression correlation analysis was performed with OncoDB (39). Correlation analysis of *ZMYND8* with the glutathione metabolic gene signature was performed with GEPIA 2 (40).

### Statistics.

Statistical analysis was performed by 2-tailed Student’s *t* test between 2 groups, and 1-way or 2-way ANOVA with multiple testing corrections within multiple groups. ELDA software was used to calculate BCSC frequency and statistical significance (41). Experiments except for ChIP-Seq that were the duplicates were repeated at least 3 times and expressed as mean ± SEM. A *P* < 0.05 was considered significant.

### Study approval.

Animal experiments were approved by the IACUC at UT Southwestern Medical Center.

### Data availability.

ChIP-Seq data were deposited at the Gene Expression Omnibus with accession numbers GSE186543, GSE203054, and GSE226834. Values for all data points in graphs are reported in the Supporting Data Values file. Raw blot data are reported in the full unedited blot and gel images file.

## Author contributions

WL and YW conceived the study, analyzed the data, and wrote the paper. ML performed most experiments, analyzed the data, and wrote the paper. LB performed ChIP-seq and NSG mouse breeding. YX assisted in flow cytometry. MZ generated plasmids. AK and CX performed bioinformatics analysis. JEW performed lentivirus production. All authors read and approved the manuscript.

## Supplementary Material

Supplemental data

Unedited blot and gel images

Supporting data values

## Version 1 01/23/2024

In-Press Preview

## Version 2 03/15/2024

Electronic publication
