# Supplementary material for: ZMYND8 protects breast cancer stem cells against oxidative stress and ferroptosis through activation of NRF2
Source: J Clin Invest. 2024 Jan 23;134(6):e171166. doi: 10.1172/JCI171166 (PMC10940091; doi:10.1172/JCI171166)
Supplement: Supplemental data [file jci-134-171166-s070.pdf]

Supplemental Data

ZMYND8 Protects Breast Cancer Stem Cells against Oxidative Stress and Ferroptosis through Activation of NRF2

Maowu Luo [1], Lei Bao [1], Yuanyuan Xue [2], Ming Zhu [1], Ashwani Kumar [3], Chao Xing [3,4], Jennifer E. Wang [1], Yingfei Wang [1,5,6,7], Weibo Luo [1,8] *

[1] Department of Pathology,
[2] Children's Medical Center Research Institute,
[3] Eugene McDermott Center for Human Growth and Development,
[4] Lyda Hill Department of Bioinformatics,
[5] Department of Neurology,
[6] Peter O'Donnell Jr. Brain Institute,
[7] Cecil H. and Ida Green Center for Reproductive Biology Sciences,
[8] Department of Pharmacology,
UT Southwestern Medical Center, Dallas, TX, USA.

Supplemental Table 1. Oligos used for generation of NRF2 KO cells.

	**Forward**	**Reverse**
KO1 sgRNA	CACCGCATACCGTCTAAATCAACAG	AAACCTGTTGATTTAGACGGTATGC
KO2 sgRNA	CACCGCACATCCAGTCAGAAACCAG	AAACCTGGTTTCTGACTGGATGTGC

Supplemental Table 2. RT-qPCR primers used in this paper.

Gene	Forward primer sequence	Reverse primer sequence	Species
GSTP1	TTGGGCTCTATGGGAAGGAC	GGGAGATGTATTTGCAGCGGA	Human
NQO1	GAAGAGCACTGATCGTACTGGC	GGATACTGAAAGTTCGCAGGG	Human
GPX2	GAATGGGCAGAACGAGCATC	CCGGCCCTATGAGGAACTTC	Human
GCLC	GGCACAAGGACGTTCTCAAGT	CAGACAGGACCAACCGGAC	Human
GCLM	CATTTACAGCCTTACTGGGAGG	ATGCAGTCAAATCTGGTGGCA	Human
TXNRD1	ATGGGCAATTTATTGGTCCTCAC	CCCAAGTAACGTGGTCTTTCAC	Human
SRXN1	CAGGGAGGTGACTACTTCTACTC	CAGGTACACCCTTAGGTCTGA	Human
SLC3A2	TGAATGAGTTAGAGCCCGAGA	GTCTTCCGCCACCTTGATCTT	Human
SLC7A11	GGTCCATTACCAGCTTTTGTACG	AATGTAGCGTCCAAATGCCAG	Human
GPX4	GAGGCAAGACCGAAGTAAACTAC	CCGAACTGGTTACACGGGAA	Human
KEAP1	CTGGAGGATCATACCAAGCAGG	GGATACCCTCAATGGACACCAC	Human
NFE2L2	TTCCCGGTCACATCGAGAG	TCCTGTTGCATACCGTCTAAATC	Human
Gstp1	ATGCCACCATACACCATTGTC	GGGAGCTGCCCATACAGAC	Mouse
Gpx2	GCCTCAAGTATGTCCGACCTG	GGAGAACGGGTCATCATAAGGG	Mouse
Gclc	GGGGTGACGAGGTGGAGTA	GTTGGGGTTTGTCCTCTCCC	Mouse
Txnrd1	CCCACTTGCCCCAACTGTT	GGGAGTGTCTTGGAGGGAC	Mouse
Srxn1	ATCGTGGTGCTGGATTGATTC	CACCCCAGAGATAAGATTACCCA	Mouse
Gclm	AGGAGCTTCGGGACTGTATCC	GGGACATGGTGCATTCCAAAA	Mouse
Slc3a2	GGTCGCGGCTAAGTTCACC	GCCCGAACGATGATAACCAC	Mouse
Slc7a11	GGCACCGTCATCGGATCAG	CTCCACAGGCAGACCAGAAAA	Mouse
Gpx4	GCCTGGATAAGTACAGGGGTT	CATGCAGATCGACTAGCTGAG	Mouse
ZMYND8	GGGTTTATCACGCTAAGTGTCTG	GGCTTTACTCTGGGTCTCGATG	Human
18sRNA	CGGCGACGACCCATTCGAAC	GAATCGAACCCTGATTCCCCGTC	Human/Mouse

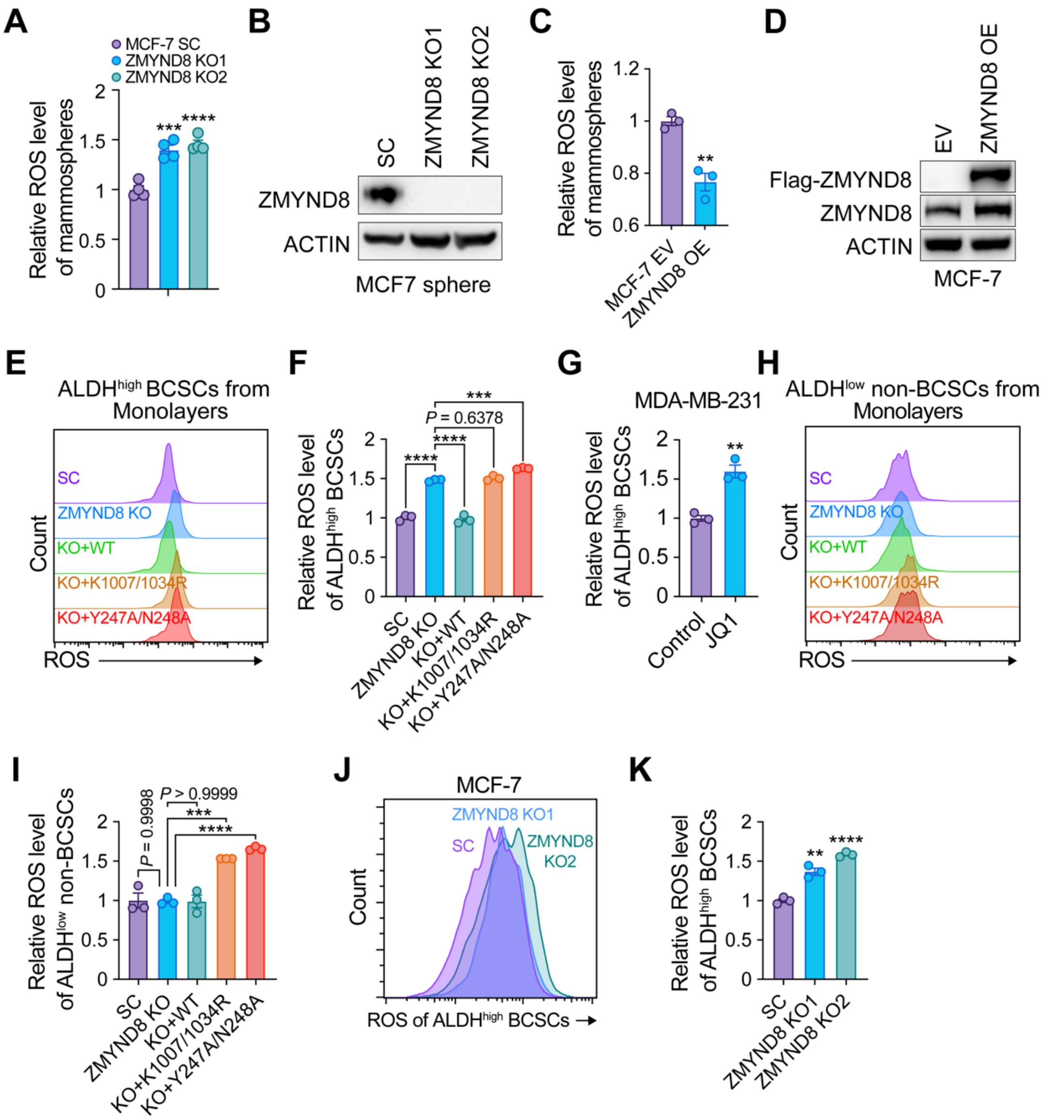

Supplemental Figure 1. Loss of ZMYND8 increases ROS in ALDH^{high} BCSCs. (**A**) Relative ROS levels in MCF-7-SC, ZMYND8 KO1, and ZMYND8 KO2 mammospheres (n = 4). (**B**) Analysis of ZMYND8 protein levels in MCF-7-SC, ZMYND8 KO1, and ZMYND8 KO2 mammospheres (n = 3). (**C**) Relative ROS levels in MCF-7 mammospheres expressing EV or WT ZMYND8 (n = 3). (**D**) Analysis of ZMYND8 protein levels in MCF-7 mammospheres expressing EV or WT ZMYND8 (n = 3). (**E and F**) Relative ROS levels in ALDH^{high} cells from MDA-MB-231-SC, ZMYND8 KO, ZMYND8 KO rescued with WT ZMYND8, K1007/1034R, or Y247A/N248A monolayers (n = 3). (**G**) Relative ROS levels in ALDH^{high} BCSCs from MDA-MB-231 cells treated with vehicle or JQ1 for four days (n = 3). (**H and I**) Relative ROS levels in ALDH^{low} non-BCSCs from MDA-MB-231-SC, ZMYND8 KO, ZMYND8 KO rescued with WT ZMYND8, K1007/1034R, or Y247A/N248A monolayers (n = 3). (**J and K**) Relative ROS levels in ALDH^{high} BCSCs from MCF-7-SC, ZMYND8 KO1, and ZMYND8 KO2 cells (n = 3). Data represent mean ± SEM. P value was determined by using one-way ANOVA corrected with Dunnett's test (**A** and **K**) or with Tukey's test (**F** and **I**), and two-tailed Student's t test (**C** and **G**). $^{**}p < 0.01$; $^{***}p < 0.001$; $^{****}p < 0.0001$.

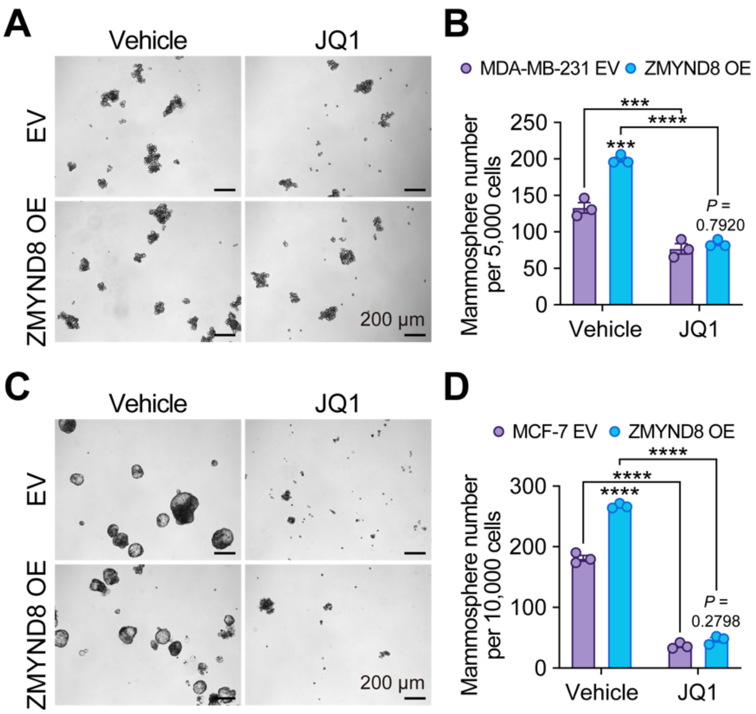

Supplemental Figure 2. Treatment of JQ1 blocks ZMYND8-induced mammosphere formation. (**A** and **B**) Formation of MDA-MB-231 mammospheres expressing EV or WT ZMYND8 treated with vehicle or JQ1. Representative mammosphere images (**A**). Quantification of mammosphere numbers (**B**, $n = 3$). (**C** and **D**) Formation of MCF-7 mammospheres expressing EV or WT ZMYND8 treated with vehicle or JQ1. Representative mammosphere images (**C**). Quantification of mammosphere numbers (**D**, $n = 3$). Data represent mean ± SEM. P value was determined using two-way ANOVA corrected with Tukey's test (**B** and **D**). ***$p < 0.001$; ****$p < 0.0001$.

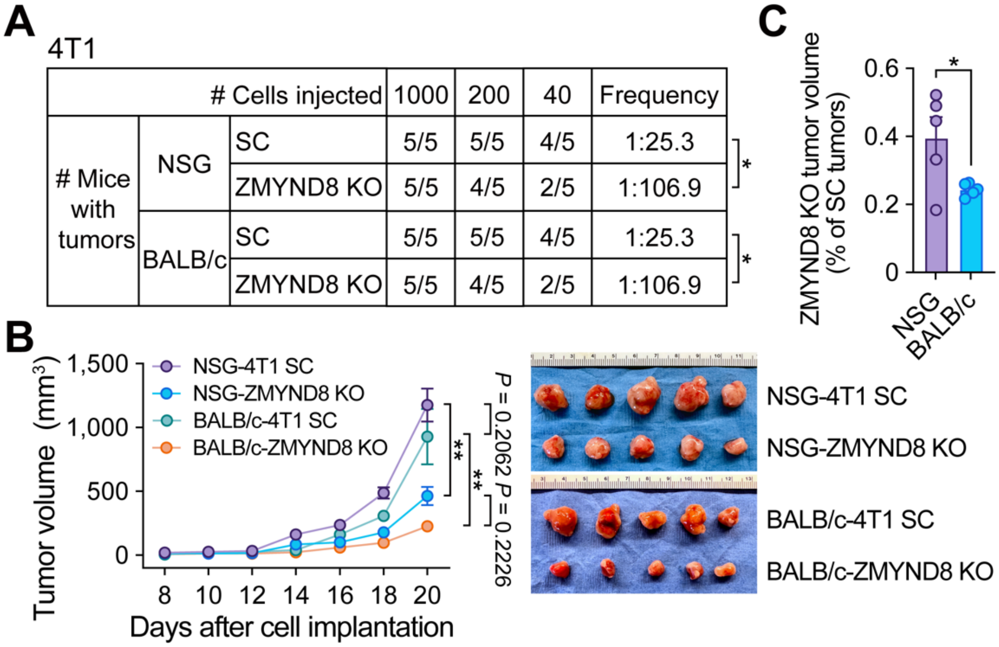

		# Cells injected	1000	200	40	Frequency	
# Mice with tumors	NSG	SC	5/5	5/5	4/5	1:25.3	*
		ZMYND8 KO	5/5	4/5	2/5	1:106.9	
	BALB/c	SC	5/5	5/5	4/5	1:25.3	*
		ZMYND8 KO	5/5	4/5	2/5	1:106.9	

Supplemental Figure 3. Effects of ZMYND8 on tumor initiation and growth in immunocompromised and immunocompetent mice. (**A**) Limiting dilution assay of SC or ZMYND8 KO 4T1 cells in NSG and BALB/c mice. (**B** and **C**) SC or ZMYND8 KO 4T1 cells were implanted into the mammary fat pad of NSG and BALB/c mice ($n = 5$), respectively. Tumor volume and image are shown (**B**). Tumor inhibition by ZMYND8 KO was quantified at post-implantation day 20 (**C**). Data represent mean ± SEM. *P* value was determined by using chi-square test (**A**), two-way ANOVA corrected with Tukey's test (**B**), and two-tailed Student's *t* test (**C**). $*p < 0.05$; $**p < 0.01$.

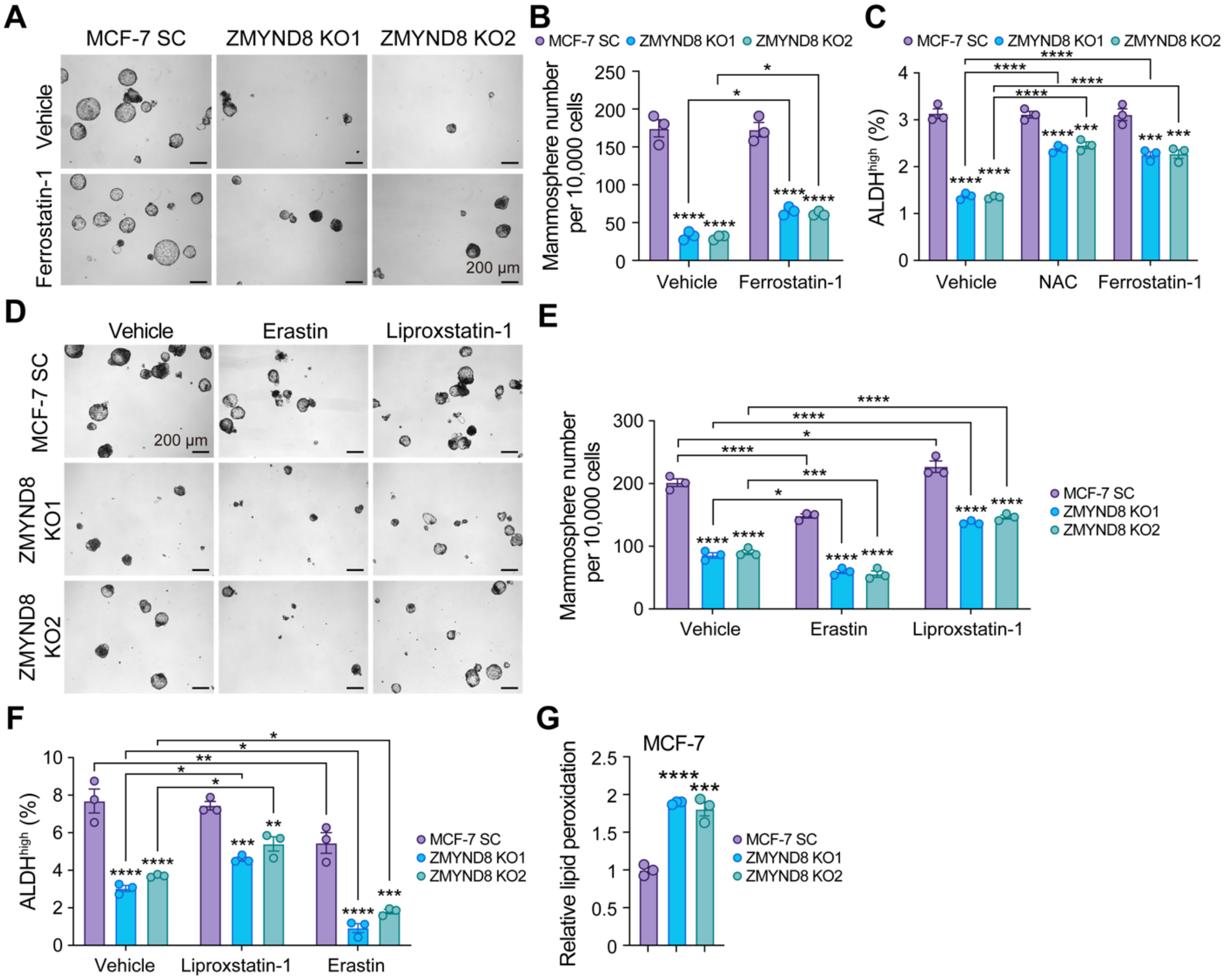

Supplemental Figure 4. ZMYND8 inhibits ferroptosis in ALDH^{high} BCSCs. (**A** and **B**) Formation of MCF-7-SC and ZMYND8 KO1/2 mammospheres treated with vehicle or Ferrostatin-1. Representative mammosphere images (**A**). Quantification of mammosphere numbers (**B**, $n = 3$). (**C**) Quantification of ALDH^{high} BCSCs in MCF-7-SC and ZMYND8 KO1/2 cells treated with vehicle, NAC, or Ferrostatin-1 ($n = 3$). (**D** and **E**) Formation of MCF-7-SC, ZMYND8 KO1, and ZMYND8 KO2 mammospheres treated with vehicle, Erastin, or Liproxstatin-1. Representative mammosphere images (**D**). Quantification of mammosphere numbers (**E**, $n = 3$). (**F**) Quantification of ALDH^{high} BCSCs in MCF-7-SC and ZMYND8 KO1/2 cells treated with vehicle, Liproxstatin-1, or Erastin ($n = 3$). (**G**) Relative lipid peroxidation levels in MCF-7-SC and ZMYND8 KO1/2 mammospheres ($n = 3$). Data represent mean ± SEM. *P* value was determined by using two-way ANOVA corrected with Tukey's test (**B**, **C**, **E**, and **F**) and one-way ANOVA corrected with Dunnett's test (**G**). $*p < 0.05$; $**p < 0.01$; $***p < 0.001$; $****p < 0.0001$.

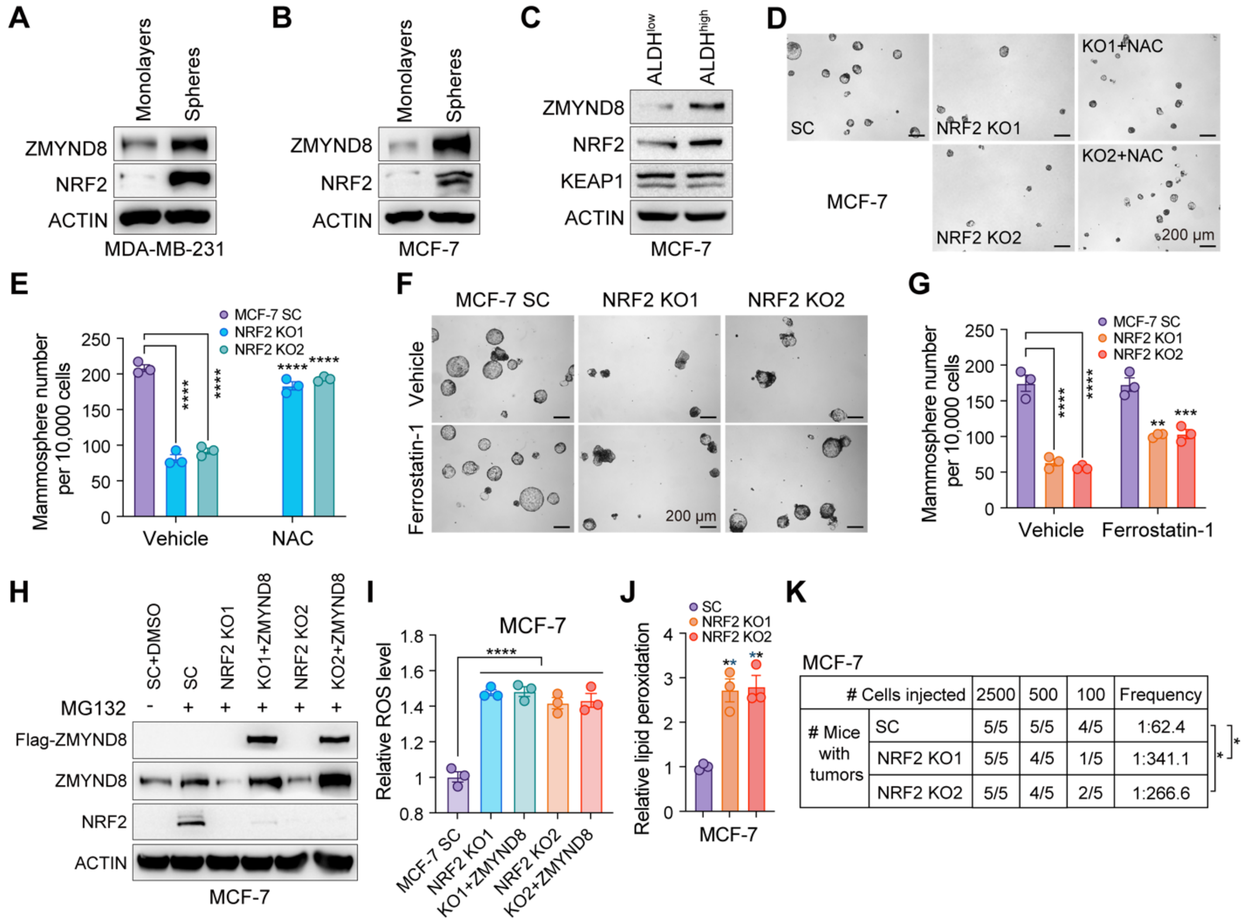

MCF-7

	# Cells injected	2500	500	100	Frequency
# Mice with tumors	SC	5/5	5/5	4/5	1:62.4
	NRF2 KO1	5/5	4/5	1/5	1:341.1
	NRF2 KO2	5/5	4/5	2/5	1:266.6

Supplemental Figure 5. ZMYND8 inhibits ferroptosis in an NRF2-dependent manner. (**A** and **B**) Analysis of ZMYND8 and NRF2 protein levels in MDA-MB-231 (**A**) and MCF-7 (**B**) mammospheres (*n* = 3). (**C**) Analysis of ZMYND8, NRF2, and KEAP1 protein levels in ALDH^{high} BCSCs and ALDH^{low} non-BCSCs from MCF-7 cells (*n* = 3). (**D** and **E**) Formation of MCF-7-SC, NRF2 KO1, and NRF2 KO2 mammospheres treated with vehicle or NAC. Representative mammosphere images (**D**). Quantification of mammosphere numbers (**E**, *n* = 3). (**F** and **G**) Formation of MCF-7-SC, NRF2 KO1, and NRF2 KO2 mammospheres treated with vehicle or Ferrostatin-1. Representative mammosphere images (**F**). Quantification of mammosphere numbers (**G**, *n* = 3). The experiments in Supplemental Figure 4A and 5F were carried out concomitantly with the same MCF-7-SC controls. (**H**) Analysis of ZMYND8 and NRF2 protein levels in MCF-7-SC, NRF2 KO1/2, NRF2 KO1/2 plus WT ZMYND8 cells treated with DMSO (-) or MG132 (+). *n* = 3. (**I**) Relative ROS levels in MCF-7-SC, NRF2 KO1/2, NRF2 KO1/2 plus WT ZMYND8 mammospheres (*n* = 3). (**J**) Relative lipid peroxidation levels in MCF-7-SC, NRF2 KO1, and NRF2 KO2 mammospheres (*n* = 3). (**K**) Limiting dilution assay of MCF-7-SC, NRF2 KO1, or NRF2 KO2 cells in NSG mice. Data represent mean ± SEM. *P* value was determined by using two-way ANOVA corrected with Tukey's test (**E** and **G**), one-way ANOVA corrected with Tukey's test (**I**) or Dunnett's test (**J**), and chi-square test (**K**). *$p < 0.05$; **$p < 0.01$; ***$p < 0.001$; ****$p < 0.0001$.

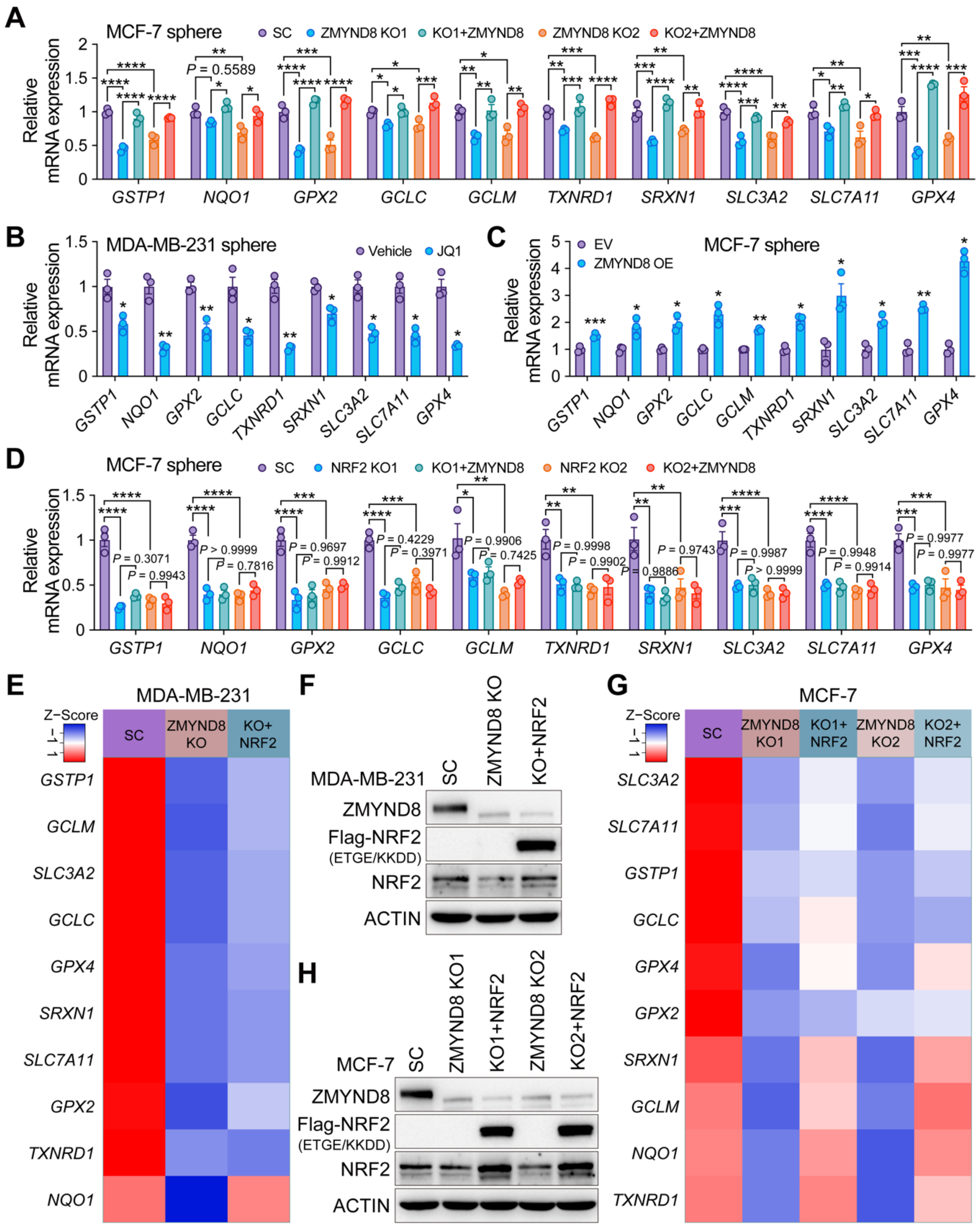

Supplemental Figure 6. ZMYND8 enhances the transcription activity of NRF2. (**A**) mRNA analysis of antioxidant genes in MCF-7-SC, ZMYND8 KO1/2, and ZMYND8 KO1/2 rescued with WT ZMYND8 mammospheres (n = 3). (**B**) mRNA analysis of antioxidant genes in vehicle- or JQ1-treated MDA-MB-231 mammospheres (n = 3). (**C**) mRNA analysis of antioxidant genes in MCF-7 mammospheres expressing EV or WT ZMYND8 (n = 3). (**D**) mRNA analysis of antioxidant genes in MCF-7-SC, NRF2 KO1/2, and NRF2 KO1/2 plus WT ZMYND8

mammospheres (n = 3). (**E** and **F**) Analysis of antioxidant gene mRNAs (**E**) and NRF2 and ZMYND8 protein levels (**F**) in MDA-MB-231-SC, ZMYND8 KO, and ZMYND8 KO plus NRF2-ETGE/KKDD mammospheres (n = 3). (**G** and **H**) Analysis of antioxidant gene mRNAs (**G**) and NRF2 and ZMYND8 protein levels (**H**) in MCF-7-SC, ZMYND8 KO1/2, and ZMYND8 KO1/2 plus NRF2-ETGE/KKDD mammospheres (n = 3). Data represent mean ± SEM. *P* value was determined using one-way ANOVA corrected with Tukey's test (**A** and **D**), and two-tailed Student's *t* test (**B** and **C**). $^{*}p < 0.05$; $^{**}p < 0.01$; $^{***}p < 0.001$; $^{****}p < 0.0001$.

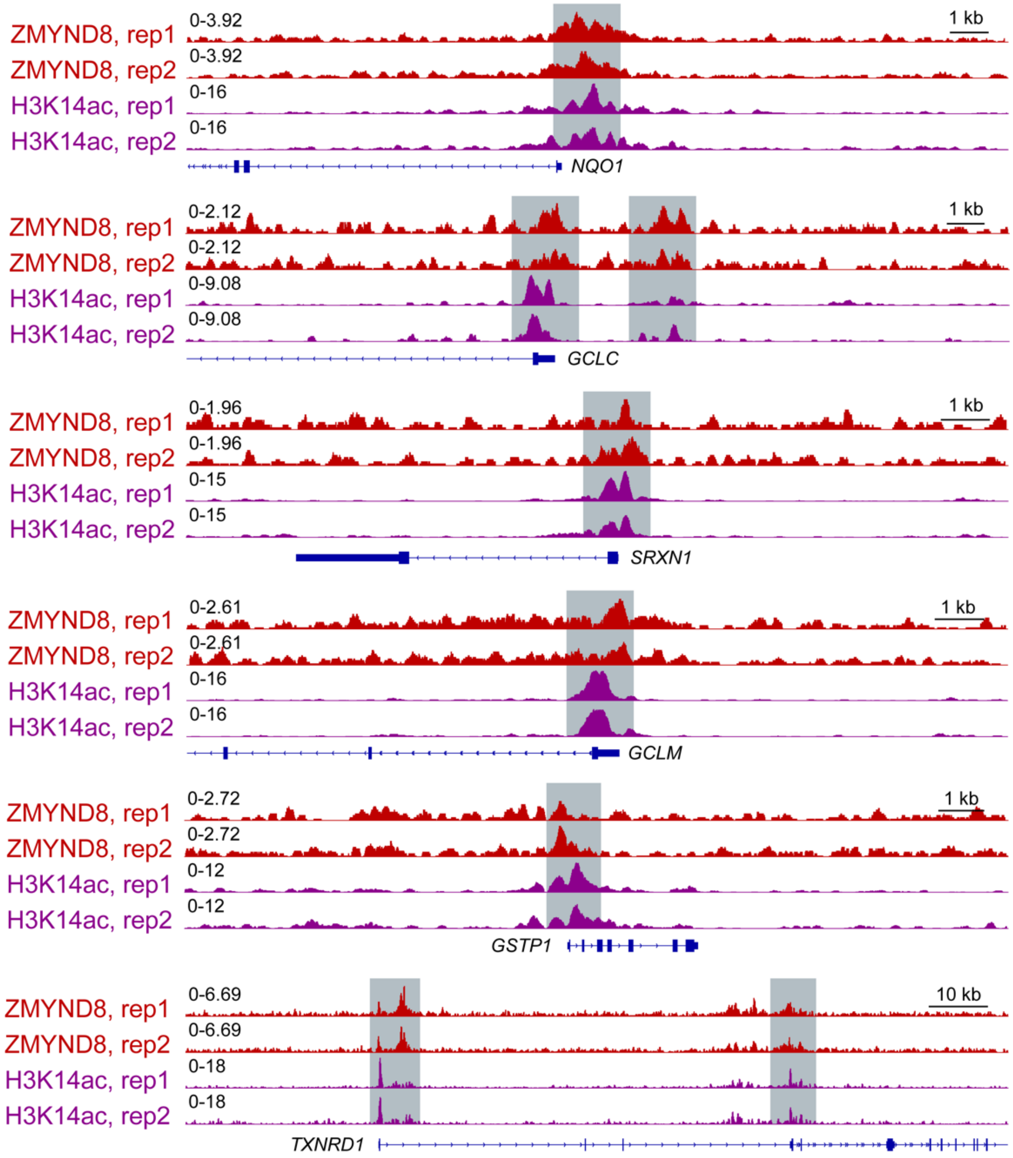

Supplemental Figure 7. Enrichment of ZMYND8 and H3K14ac on NRF2 target genes. Genome browser snapshots of ZMYND8 and H3K14ac ChIP-seq peaks on antioxidant genes. rep, replicate.

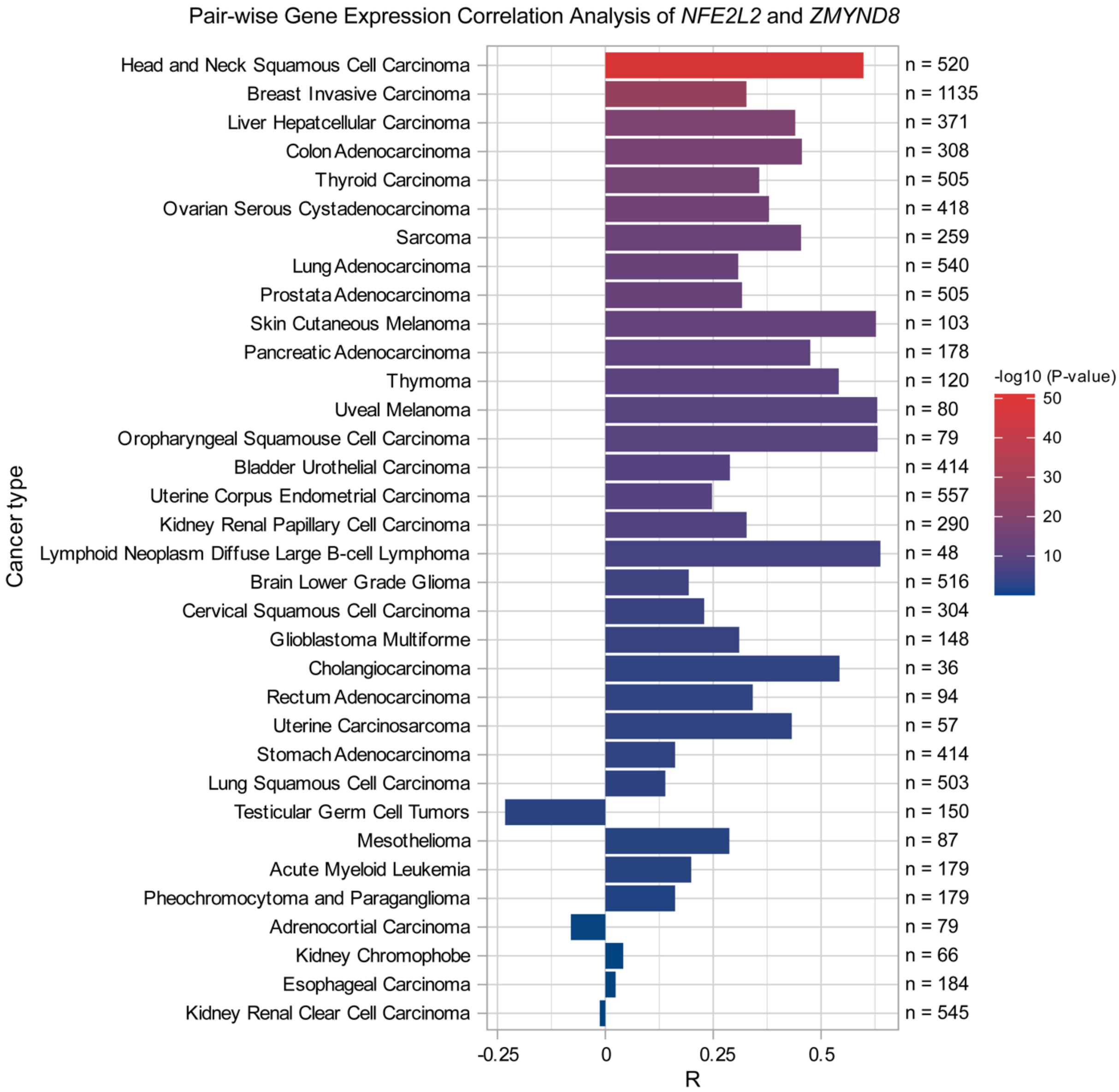

Supplemental Figure 8. *ZMYND8* is positively co-expressed with *NFE2L2* in human tumors. Pair-wise gene expression correlation analysis of *NFE2L2* and *ZMYND8* in human tumors from TCGA cohort (OncoDB, $P < 0.05$ was considered as correlation).
