# Supplementary material for: ZMYND8 protects breast cancer stem cells against oxidative stress and ferroptosis through activation of NRF2
Source: J Clin Invest. 2024 Jan 23;134(6):e171166. doi: 10.1172/JCI171166 (PMC10940091; doi:10.1172/JCI171166)

Full unedited gel for Figure 1

Figure 1B

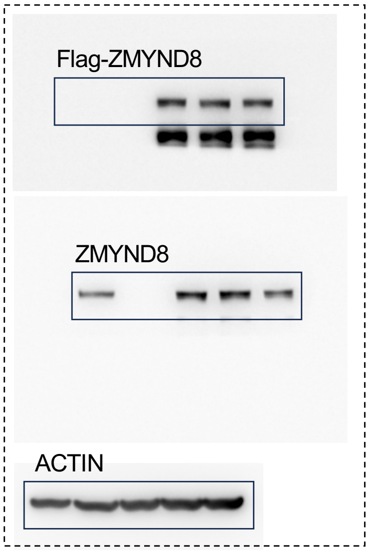

Figure 1D

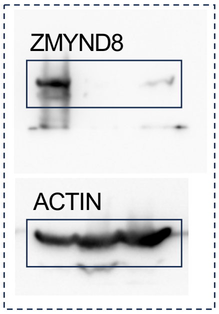

Figure 1F

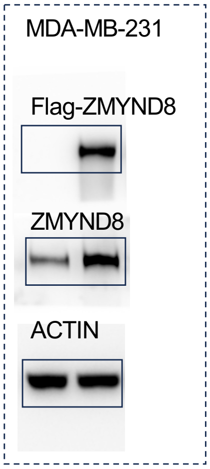

Figure. 1K

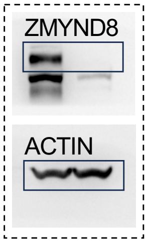

Full unedited gel for Figure 4

Figure. 4A

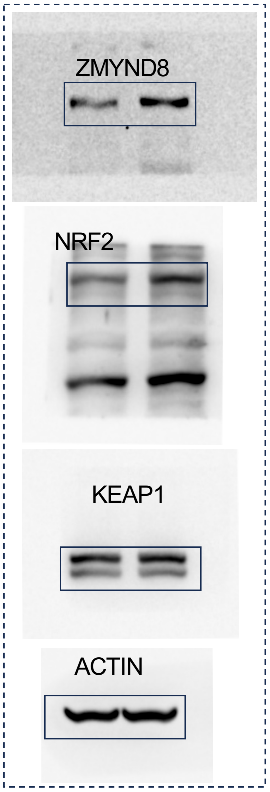

Figure. 4F

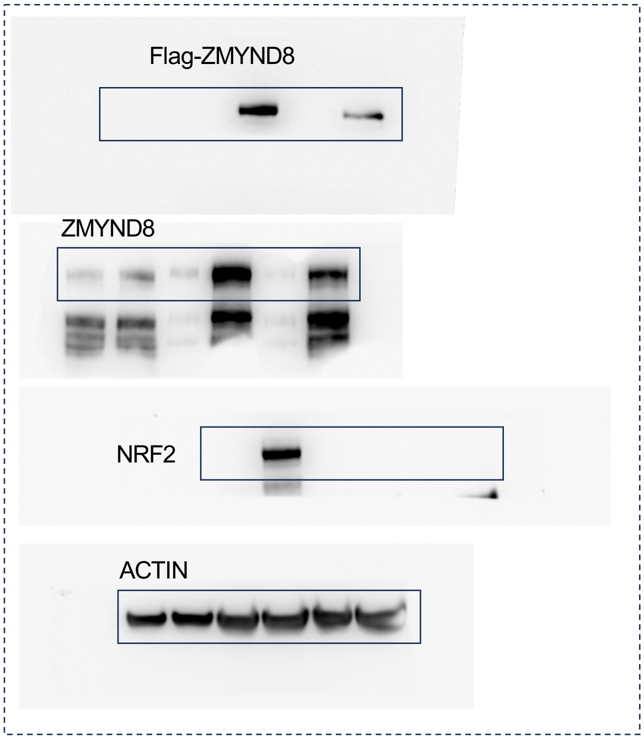

Full unedited gel for Figure 5

Figure 5A

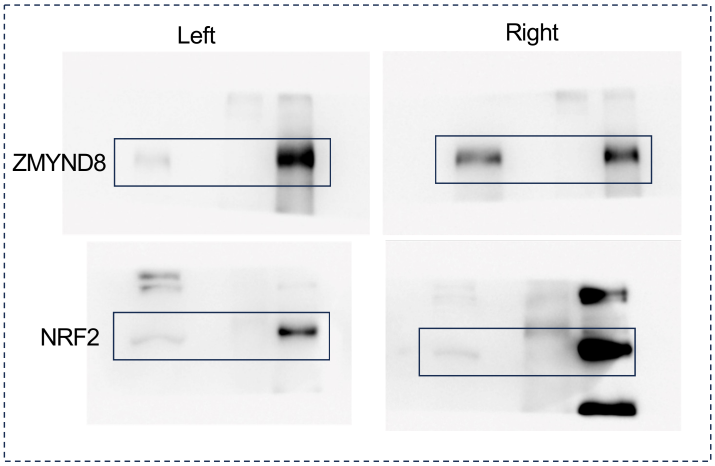

Figure 5B

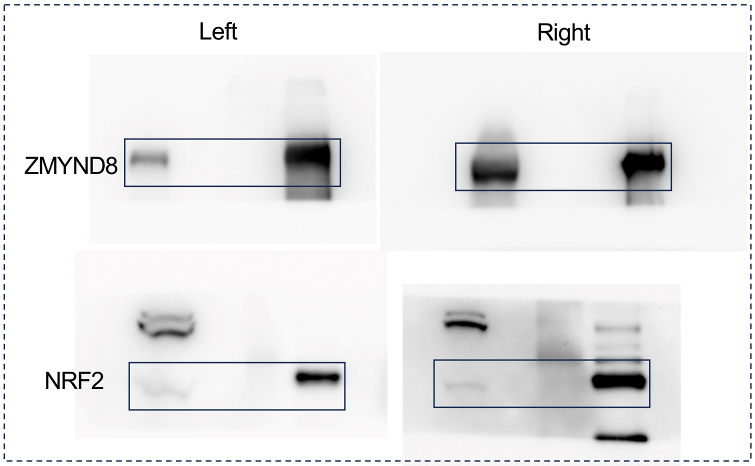

Full unedited gel for Figure 5

Figure 5D

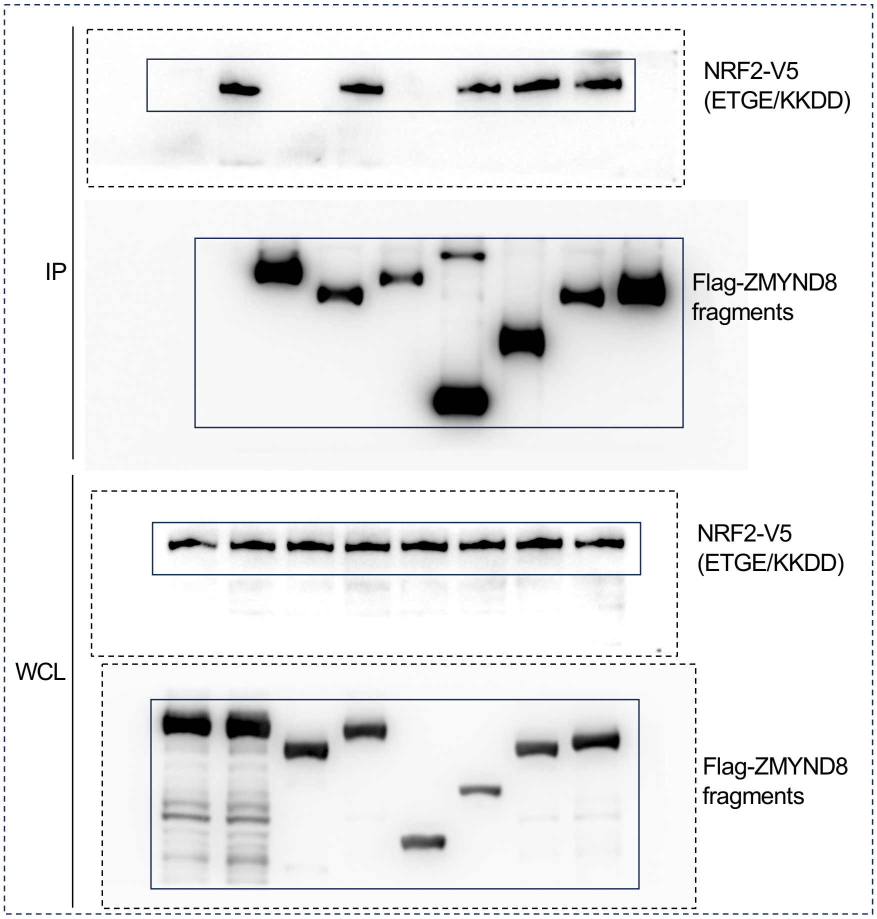

Full unedited gel for Figure 5

Figure 5F

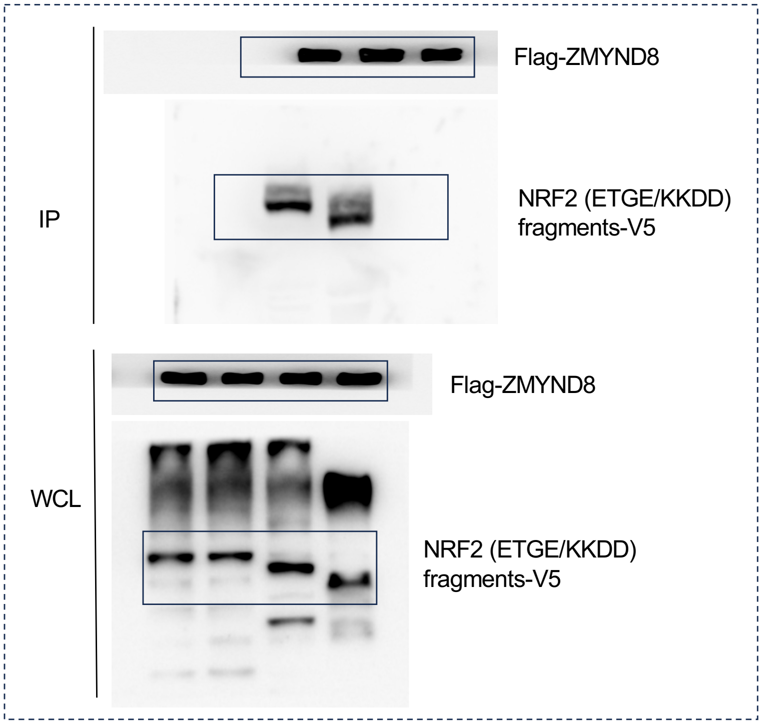

Figure 5H

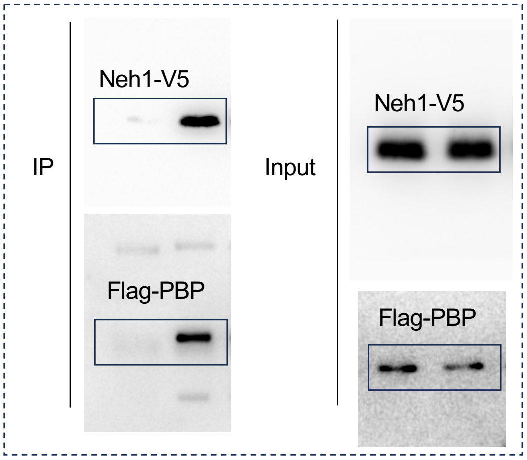

Figure 5I

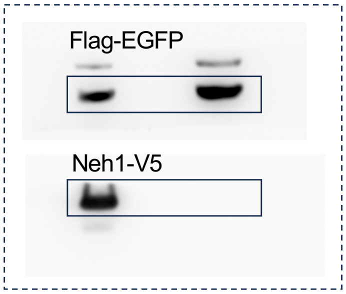

Full unedited gel for Figure 6

Figure 6B

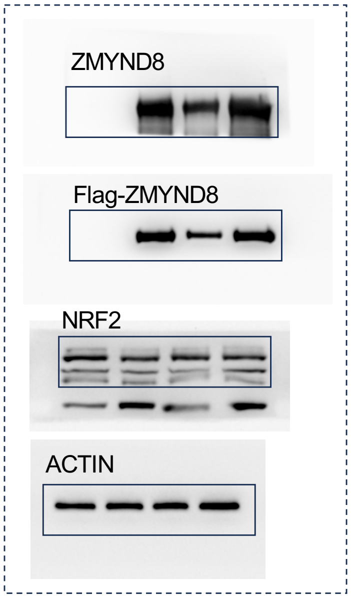

Full unedited gel for Figure 8

Figure 8A

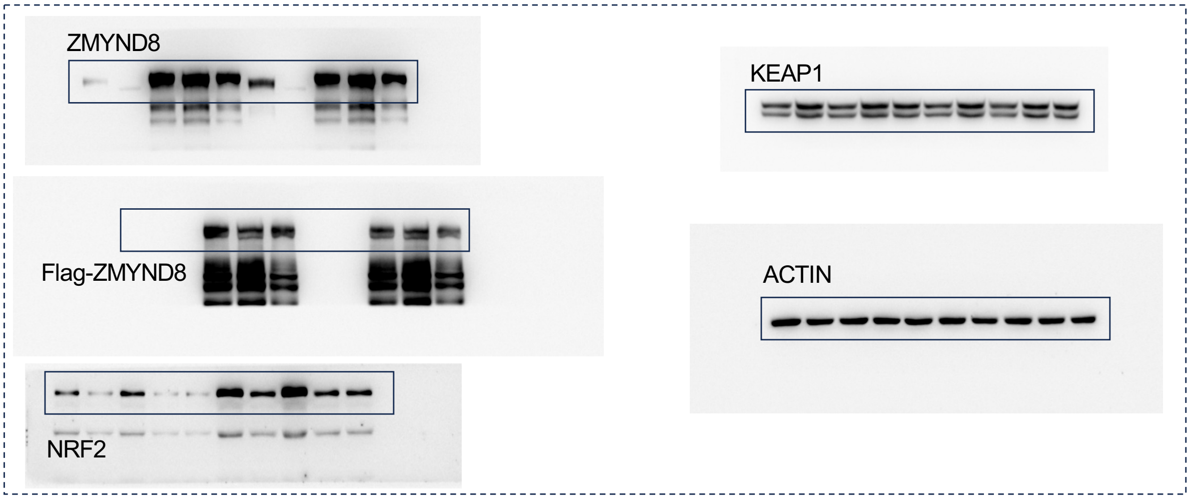

Figure 8B

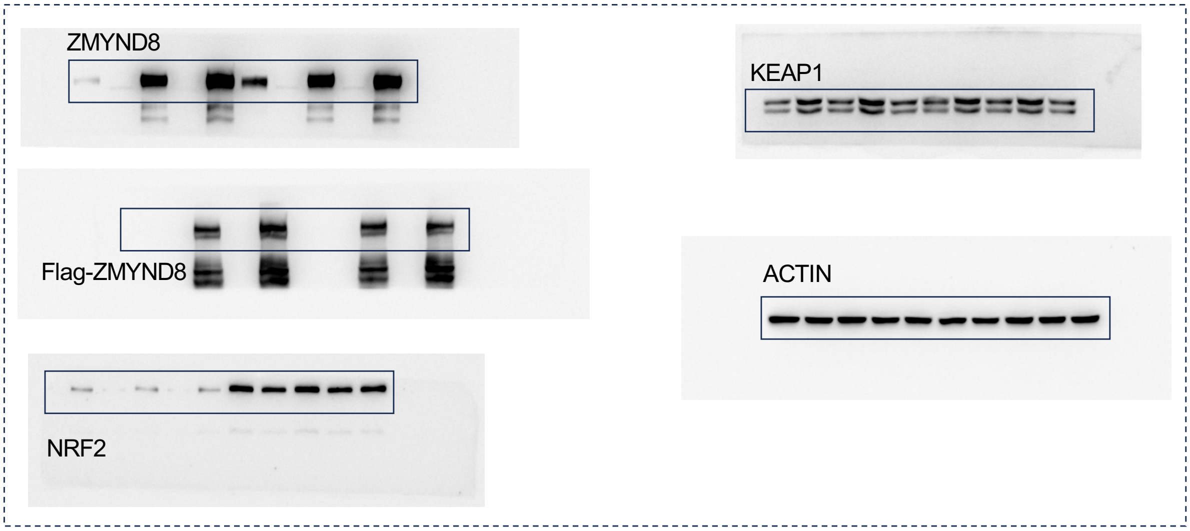

Figure 8C

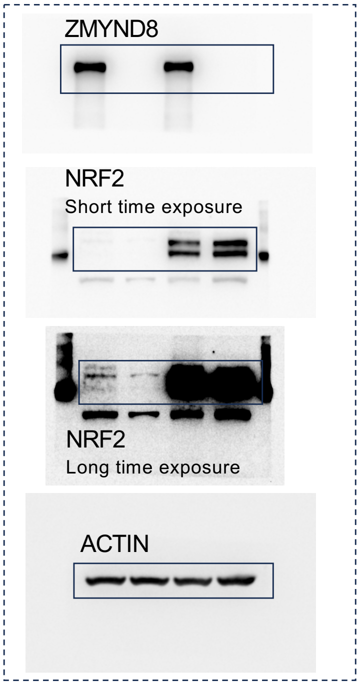

Figure 8D

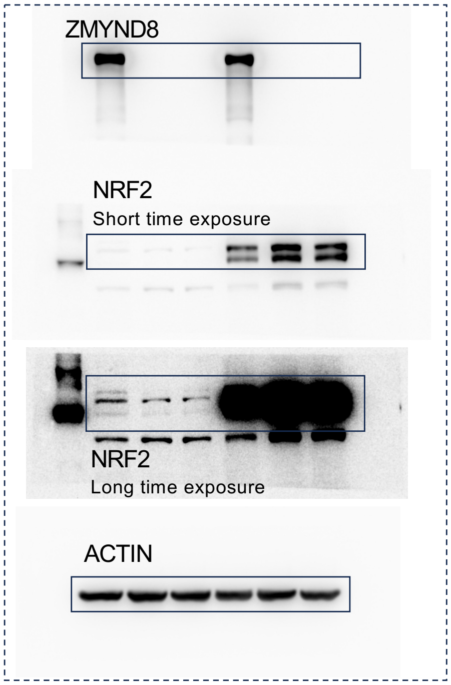

Full unedited gel for Figure 9

Figure 9D

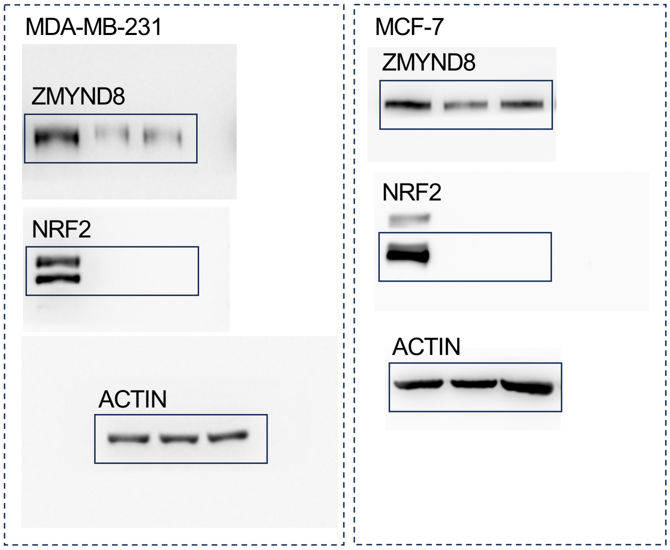

Figure 9G

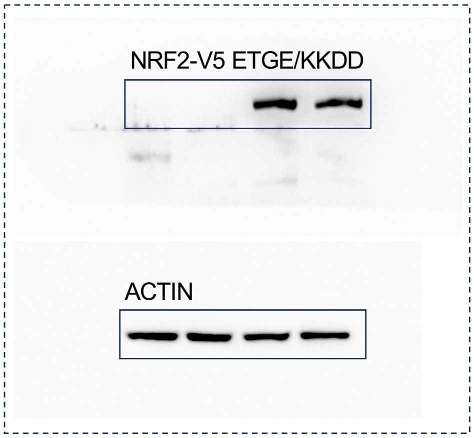

Full unedited gel for Supplemental Figure 1

Supplemental Figure 1B

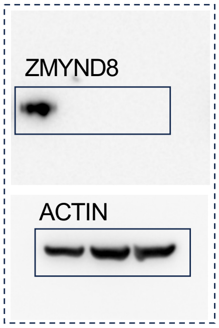

Supplemental Figure 1D

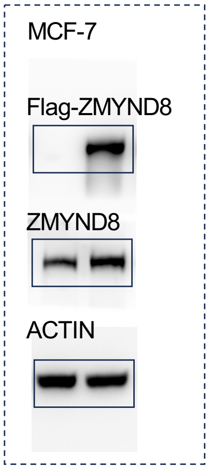

Full unedited gel for Supplemental Figure 5

Supplemental Figure 5A

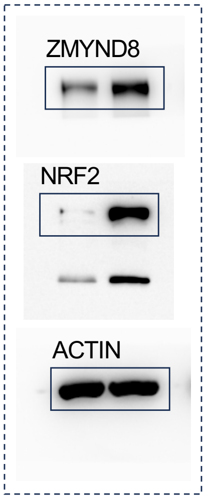

Supplemental Figure 5B

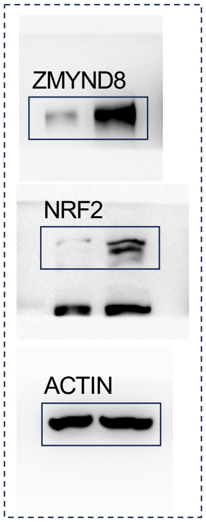

Supplemental Figure 5C

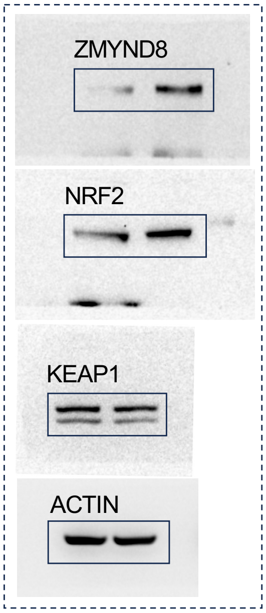

Supplemental Figure 5H

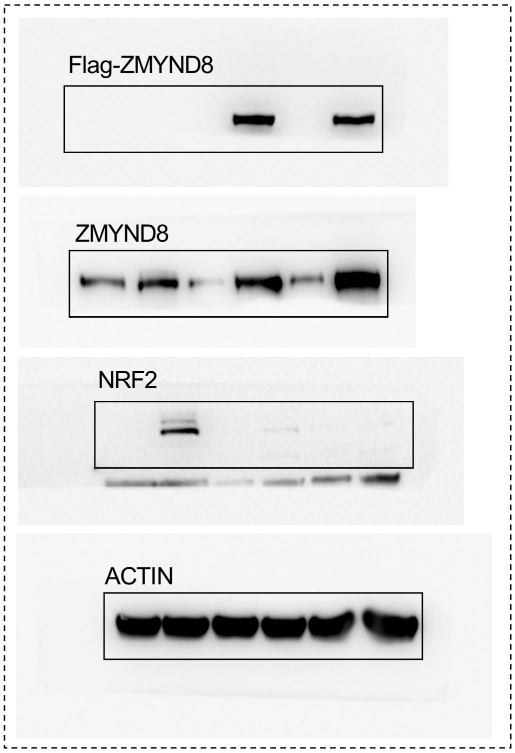

Full unedited gel for Supplemental Figure 6

Supplemental Figure 6F

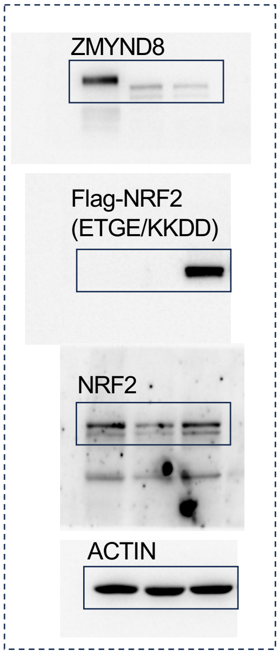

Supplemental Figure 6H

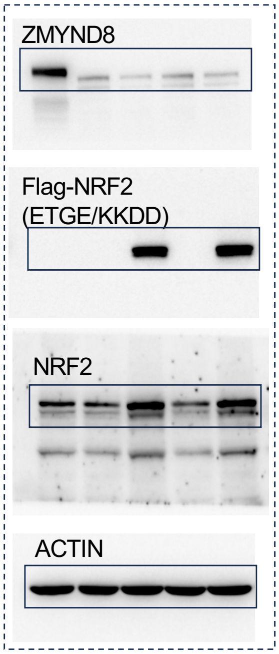

Supplement: Unedited blot and gel images [file jci-134-171166-s071.pdf]
